# Hypoglycemic Effect of Prolamin from Cooked Foxtail Millet (*Setaria italic*) on Streptozotocin-Induced Diabetic Mice

**DOI:** 10.3390/nu12113452

**Published:** 2020-11-11

**Authors:** Yongxia Fu, Ruiyang Yin, Zhenyu Liu, Yan Niu, Erhu Guo, Ruhong Cheng, Xianmin Diao, Yong Xue, Qun Shen

**Affiliations:** 1 Key Laboratory of Plant Protein and Grain Processing, National Engineering Research Center for Fruits and Vegetables Processing, College of Food Science and Nutritional Engineering, China Agricultural University, Beijing 100083, China; fyx2012314095@163.com (Y.F.); ruiyang_yin@163.com (R.Y.); sculzy2018@163.com (Z.L.); xueyong@cau.edu.cn (Y.X.); 2 Shan Xi Dongfang Wuhua Agricultural Technology Co. Ltd., Datong 037000, China; niu_yan11@aliyun.com; 3 Research Institute of Millet, Shanxi Academy of Agricultural Sciences, Taiyuan 030031, China; guoerhu2003@163.com; 4 Research Institute of Millet, Hebei Academy of Agriculture and Forestry Sciences, Shijiazhuang 050035, China; rhcheng63@126.com; 5 Institute of Crop Science, Chinese Academy of Agricultural Sciences, Beijing 100081, China; diaoxianmin@caas.cn

**Keywords:** foxtail millet, gut microbiota, metabolomics, prolamin, type 2 diabetes

## Abstract

Millet proteins have been demonstrated to possess glucose-lowering and lipid metabolic disorder modulation functions against diabetes; however, the molecular mechanisms underlying their anti-diabetic effects remain unclear. The present study aimed to investigate the hypoglycemic effect of prolamin from cooked foxtail millet (PCFM) on type 2 diabetic mice, and explore the gut microbiota and serum metabolic profile changes that are associated with diabetes attenuation by PCFM. Our diabetes model was established using a high-fat diet combined with streptozotocin before PCFM or saline was daily administrated by gavage for 5 weeks. The results showed that PCFM ameliorated glucose metabolism disorders associated with type 2 diabetes. Furthermore, the effects of PCFM administration on gut microbiota and serum metabolome were investigated. 16S rRNA gene sequencing analysis indicated that PCFM alleviated diabetes-related gut microbiota dysbiosis in mice. Additionally, the serum metabolomics analysis revealed that the metabolite levels disturbed by diabetes were partly altered by PCFM. Notably, the decreased D-Glucose level caused by PCFM suggested that its anti-diabetic potential can be associated with the activation of glycolysis and the inhibition of gluconeogenesis, starch and sucrose metabolism and galactose metabolism. In addition, the increased serotonin level caused by PCFM may stimulate insulin secretion by pancreatic β-cells, which contributed to its hypoglycemic effect. Taken together, our research demonstrated that the modulation of gut microbiota composition and the serum metabolomics profile was associated with the anti-diabetic effect of PCFM.

## 1. Introduction

Type 2 diabetes mellitus (T2DM), characterized by hyperglycemia and insulin resistance, has become a global metabolic disease [1]. Genetic factors combined with environmental factors, including diet and lifestyle, may trigger T2DM [2,3,4]. Among these factors, unhealthy eating habits, including excess intakes of sugar, oil and flour, can lead to obesity, a major metabolic risk factor for T2DM. In contrast, an increased intake of whole grains has been proven to be strongly correlated with a lower risk of T2DM [5,6]. As such, the replacement of refined carbohydrate with whole grains is preferable for people, especially diabetic patients, in daily life.

Millet, one kind of whole grains, has received much attention for its effects on metabolic diseases, including the prevention and mitigation of diabetes and obesity [7,8,9]. The main components of millet, such as phytochemicals, fibers and proteins, may account for its beneficial effects on diabetes and obesity [7,10,11,12]. It is worth noting that millet proteins have been understudied compared to phytochemicals and fibers in diabetes mitigation. Millet proteins are mainly composed of four proteins, including prolamin, gluten, albumin, and globulin. Among these, prolamin is the major storage protein in millet. So far, several studies have reported the anti-diabetic potentials of millet prolamins. For instance, prolamin in Japanese millet protein was responsible for its hypolipidemic and hypoglycemic effects on type 2 diabetic mice [13]. Besides, peptides from prolamin in millet grains showed high pancreatic lipase inhibitory activity and lipoxygenase inhibitory activity, and thus may alleviate inflammatory responses and reduce triglyceride (TG) accumulation in obesity, and then lower T2DM incidence [14]. In addition, foxtail millet (FM) prolamin-derived peptides, proline–phenylalanine–leucine–phenylalanine and isoleucine–alanine–leucine–leucine–isoleucine–proline–phenylalanine, possess antioxidizing effects, which may be beneficial to the alleviation of oxidative stress caused by diabetes or obesity [15]. However, the current studies mainly focus on the functions of prolamin in raw millets, while grains, including millets, are usually consumed after cooking. Cooking can induce disulfide bonds and hydrophobic aggregates formation, as well as secondary structure alteration in proteins (i.e., conversion between α-helical and β-sheet structures) [16,17,18]. All of the above factors may contribute to the changes in the function of prolamin from cooked millets. Therefore, in this study, we investigated the hypoglycemic effect of prolamin from cooked FM (PCFM), a major crop cultivated mainly in Asia and Africa and ranked sixth in the world production of grains [19].

Plant proteins are hydrolyzed into short peptides or amino acids by the digestive system, absorbed in the human intestine efficiently, and then transported into body fluids to play physiological roles through circulation, and thus regulate metabolite abundance in the blood [20]. In particular, gut microbiota in the small and large intestine also play a role in dietary protein metabolism, including promoting protein digestion to produce short peptides and amino acids, which are then fermented to exert regulatory effects back on the gut microbiota [21,22]. Therefore, microbiota in the gut and metabolites in the blood can be directly or indirectly altered by digested proteins. In addition, T2DM has been demonstrated to induce gut microbiota dysbiosis. So far, the hypolipidemic effects of several cereal proteins, including rice endosperm protein and mung bean protein isolates, have been proven to be associated with the modulation of gut microbiota, which contributes to the alleviation of glucose metabolism disorders and the reduction of diabetes incidence [23,24,25]. Thus we hypothesized that oral PCFM administration may induce gut microbiota changes so as to affect diabetes development. In addition, diabetes could induce metabolic disorders that may manifest as serum metabolite alterations [26]. Dietary intervention could achieve hypoglycemic effects by repairing serum metabolite disturbances caused by diabetes [27]. Metabolomics in body fluids, such as blood and urine, has been utilized to find out potential biomarkers and then reveal the responses of diet supplementation to diabetes [28]. However, the contribution of metabolomic changes induced by millet protein to diabetes treatment is unclear. Taken together, we proposed that PCFM ingestion can lead to alterations in gut microbiota and serum metabolites, and therefore facilitate blood glucose regulation.

In this study, gut microbiota and serum metabolomics biomarkers related to the hypoglycemic effect of PCFM were evaluated using 16S rRNA gene sequencing and ultra-high-performance liquid chromatography coupled with quadrupole time-of-flight mass spectrometry (UPLC-Q-TOF/MS), respectively. We demonstrated that PCFM could ameliorate glucose homeostasis disorders and alleviate TG accumulation in diabetic mice. Gut microbiota composition alteration and serum metabolic profile modulation were associated with the hypoglycemic effect of PCFM.

## 2. Materials and Methods

### 2.1. Preparation of PCFM

FM (variety: Dongfangliang) was provided by Shanxi Dongfang Wuhua Agricultural Technology Co., LTD. (Shanxi, China). 

Preparation of cooked FM flour: FM was ground to flour using a laboratory grinder (Jiupin Industry and Trade Co., Ltd., Zhejiang, China) and then sieved through a 60-mesh screen. Then, the FM flour was dispersed into n-hexane at a ratio of 1:5 (w/v) and stirred in a water bath at 37 °C for 4 h. After standing for 30–60 min at room temperature, the FM was collected by discarding the n-hexane and drying in a hot air oven to entirely remove the hexane. The resultant flour was the raw FM. Subsequently, raw FM was mixed with distilled water at a ratio of 1:5, which was then reacted in a boiling water bath for 10 min and centrifuged at 10,000 rpm for 10 min to obtain the precipitate. The precipitate was dried in a hot air oven at 43 °C for 24 h to obtain the cooked FM flour, which was stored at −20 °C for use.

Extraction of PCFM: The cooked FM flour was dispersed in 70% ethanol solution at a ratio of 1:7 (w/v), vibrated in a constant temperature bath oscillator at 37 °C for 2 h and centrifuged at 8000 rpm for 15 min to collect the supernatant. Then, the supernatant was dialyzed using dialysis bags of Shanghai Yuanye Biotechnology Co., Ltd. (Shanghai, China) for 24 h. The distilled water for dialysis was replaced at least 4 times. At the end of dialysis, the dialysate was centrifuged at 7000 rpm for 5 min to obtain the precipitate. Then, the precipitate was lyophilized by a vacuum freeze drier (Biocool, Beijing, China) to obtain the PCFM. The protein content of PCFM was 85%.

### 2.2. Animal Experimental Design

**Figure 1:**
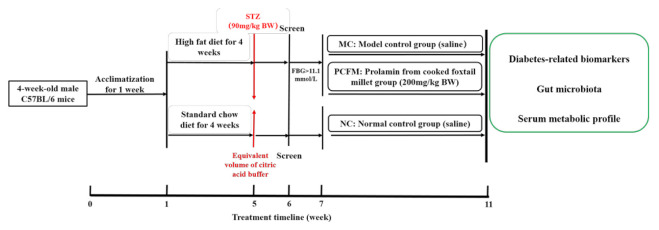
Experimental design. After acclimatization for 1 week, 5-week-old male C57BL/6J mice were fed with a high-fat diet (HFD) or standard chow diet for 4 weeks. At the 5th week, male C57BL/6J mice were injected with streptozotocin (STZ) intraperitoneally at the dose of 90 mg/kg body weight (BW). One week after STZ injection, fasting blood glucose (FBG) was measured and mice with FBG levels > 11.1 mmol/L were considered as diabetic mice, and randomly allocated to the model control group (MC, administrated with saline by oral gavage) or the prolamin from cooked foxtail millet group (PCFM, administrated with 200 mg/kg BW PCFM by oral gavage). After five weeks’ treatment, the effects of PCFM on the diabetes-related biomarkers, gut microbiota and serum metabolic profiles of diabetic mice were evaluated.

Male C57BL/6 mice weighing 20 ± 1 g and aged 4 weeks were provided by Beijing Vital River Laboratory Animal Technology Co., Ltd (Beijing, China). All mice were fed in a controlled environment (temperature 22 ± 1 °C; humidity 60 ± 10%; a 12 h/12 h light/dark cycle) with free access to water and diet. All experimental procedures were approved by the Animal Care Committee of China Agricultural University (approval number: AW01089102-4). The experimental design is presented in Figure 1. After acclimatization for 1 week, 8 mice were randomly allocated to normal control group (NC) and fed with standard chow diet (SCD, D12450J, 10% fat). The other mice (*n* = 22) were fed with a high-fat diet (HFD, D12492, 60% fat). All feeds were supplied by Research Diets, Inc. (New Brunswick, NJ, USA). After being fed with the HFD for 4 weeks, the diabetes model was induced by intraperitoneal injection of 90 mg/kg body weight (BW) streptozotocin (STZ) at the 5th week [29]. STZ was purchased from Sigma Aldrich (St. Louis, MO, USA). Mice in the NC group were injected with an equivalent volume of citric acid buffer. Fasting blood glucose (FBG) was measured one week after STZ injection. Briefly, mice were fasted for 6 h and blood samples were collected from the tail vein, and the FBG levels were determined using a glucose meter (Byer, Ascensia Diabetes Care Shanghai Co., Ltd., Shanghai, China). Mice with FBG > 11.1 mmol/L in the HFD group were selected as diabetic mice [30,31]. Diabetic mice were randomly divided into the model control group (MC, physiological saline, *n* = 8) and PCFM group (PCFM, 200 mg/kg BW, *n* = 8) with a precondition that there was no significant difference in the FBG levels between the MC and PCFM groups before treatment. Mice in the NC group were administered with saline. That is, from the 7th to 11th week of the experiment, all mice were administrated with physiological saline or PCFM by gavage once per day, and mice in the MC and PCFM group were still fed with HFD. Notably, PCFM was suspended in physiological saline to obtain solutions with a concentration of 30 mg/mL. The gavage volume of PCFM in each mouse was varied according to their BW and the maximum gavage volume was no more than 0.3 mL. The mice in the NC and MC groups were orally administrated with an equivalent volume of saline. The gauge of the syringe used in our study was 1 ml. Curved stainless steel gavage needles with a length of 5.5 cm were chosen to match the syringes. The BW of mice was recorded every week. After 5 weeks’ treatment, an oral glucose tolerance test (OGTT) was performed as follows. Briefly, mice were fasted for 6 h and 1 g/kg BW glucose solution was injected into mice by gavage, and blood glucose (BG) was measured at 0 min, 30 min, 60 min and 120 min [32]. The area under curve (AUC) over 120 min was calculated following the trapezoidal rule.

AUC = 0.25 (BG_0_ + 2BG_30_) +0.5 (1.5BG_60_ + BG_120_).

BG_0_, BG_30_, BG_60_ and BG_120_= BG levels at 0, 30, 60 and 120 min after glucose administration.

BW gain was calculated by the difference in body weight at the 7th and 11th weeks of the experiment. Three days after performing the OGTT, all mice were fasted for 6 h. Then blood samples were collected via orbital venous plexus and frozen in liquid nitrogen for serum metabolomics analysis. Then the mice were sacrificed and dissected, and liver samples were collected via forceps, rinsed with physiological saline, and then frozen in liquid nitrogen. In addition, fecal samples were also collected and frozen in liquid nitrogen for 16S rRNA gene sequencing.

### 2.3. Biochemical Analysis

Diabetes development evaluation, including fasting insulin, aspartate aminotransferase (AST), alanine aminotransferase (ALT) and TG in serum and liver, were measured using kits from the Nanjing Jiancheng Bioengineering Institute (Nanjing, China). The homeostasis model assessment of β cell function (HOMA-β) and the homeostasis model assessment of insulin resistance (HOMA-IR) were calculated as follows: HOMA-β = (20 × Fasting Insulin (mU/mL))/((FBG (mM) − 3.5)); HOMA-IR = (FBG (mM) × fasting insulin (mU/L))/22.5 [33].

### 2.4. Gut Microbiota Analysis

Feces samples were collected and frozen in liquid nitrogen. Genomic DNA was extracted from feces and DNA quality was checked by 1% agarose gel electrophoresis (Beijing Liuyi Biotechnology Co., Ltd., Beijing, China). The V3-V4 region of the bacteria 16S rRNA gene was amplified using the primers 338F/806R (338F: 5′-ACTCCTACGGAGGCAGCAGCAG-3′; 806R: 5′-GGACTACHVGGTWTCTAAT-3′) by PCR (Applied Bio-systems, Waltham, MA, USA) [34,35]. Then, PCR products of the same sample were mixed, extracted by 2% agarose gel electrophoresis, and purified using an AxyPrep DNA Gel Extraction Kit (Axygen Biosciences Inc., Union City, CA, USA). Notably, Tris-Acetate (TAE) buffer was used in gel electrophoresis. The gel was run at a voltage of 100 V for 30 min. The PCR products were quantified by a Quanti FluorTM-ST blue fluorescence quantitative system (Promega Corporation, Madison, WI, USA). Then Miseq library was constructed and sequenced by a TruSeq™ DNA Sample Prep Kit (Illumina Inc., San Diego, CA, USA). The quality of the sequence was controlled and filtered using QIIME (Version 1.9.1, University of Colorado, Boulder, CO, USA), and then operational taxonomic units (OTUs) at a 97% similarity level were identified using USEARCH (Version 7.0, developted by Edgar, R.C., who is currently an independent researcher). In order to obtain the species information at different classification levels (i.e., kingdom, phylum, class, order, family, genus, species) corresponding to each OTU, the representative sequence in each OTU was picked out, and a Ribosomal Database Project classifier based on the Bayes algorithm was used to compare the representative sequence of each OTU with the Silva (SSU123) 16S rRNA database to find the species with a 70% reliability at different classification levels. Then, we determined all bacteria abundances at different classification levels in each sample. Thus the relative abundance of each bacterium referred to the percent of a specific bacterium’s abundance in comparison to the sum of all bacteria abundance at the same classification level. The α diversity, including rarefaction curves (comparing the species richness of different samples and explaining whether the sampling numbers were reasonable) and Shannon index (estimating the level of community diversity), were analyzed by Mothur (Version 1.30.2, The University of Michigan, Ann Arbor, MI, USA). In the present study, the rarefaction and Shannon index curves tended to be flat, thus revealing that the amount of sequencing data was large enough to reflect the vast majority of microbial information in all samples (Figure S1). The distance calculation of β diversity, including principal coordinates analysis (PCoA) and nonmetric multidimensional scaling (NMDS), was performed based on Bray–Curtis. A Wilcoxon rank sum test was used to compare the significance of the relative abundance differences among different groups. The linear discriminant analysis (LDA) with a threshold of 3 was performed to identify the characteristic bacteria at genus level associated with PCFM supplementation. 

### 2.5. Unbiased Metabolomics Analysis

At the end of the experiment, blood samples were collected, centrifuged (3000 rpm, 15 min) to obtain serum samples, and then stored in −80 °C for further analysis. Serum metabolomics analysis using UPLC-Q-TOF/MS was performed by Majorbio Bio-Pharm Technology Co., Ltd (Shanghai, China). Briefly, 100 µL measurements of serum samples were accurately measured, and 20 µL of internal standard (0.3 mg/mL, prepared by L-2-chloro-phenylalanine and acetonitrile) was mixed evenly with the serum samples. Then 400 µL of extraction solution (methanol: acetonitrile = 1:1) was added followed by extraction through ultrasonic at 5 °C, 40 KHz for 30 min. After being left at −20 °C for 30 min, the serum samples were centrifuged at 4 °C (11,766 rpm, 15 min) and the supernatant was collected and dried. Then, 100 µL of the duplicate solution (acetonitrile: water = 1:1) was added to re-dissolve the dried samples. Finally, the samples were transferred to the injection vials for LC-MS analysis.

Metabolomics analysis was performed using a UPLC–Triple TOF system of UPLC-TOF-MS (AB SCIEX, Framingham, MA, USA). Separation was carried out on a HSS T3 column (100 mm × 2.1 mm, 1.8 µm; Waters, Milford, MA, USA; kept at 40 °C) and the flow rate was 0.40 mL/min. Aqueous formic acid (0.1%, v/v) (A) and acetonitrile/isopropanol (1:1, v/v) (containing 0.1% formic acid, v/v) (B) were used as mobile phases. The gradient elution of B was performed as follows: 5–20% B at 0–3 min, 20–95% B at 3–9 min, 95% B at 9–13 min, 95–5% B at 13.0–13.1 min, and then maintained at 5% B for 2.9 min. The injection volume of samples was 10 μL. The electrospray ionization (ESI) source in both positive and negative ion modes was used in MS analysis, and ion spray voltage was used for mass spectrometer calibration. MS data were collected in a mass range of 50–1000 Da. Then the UPLC-TOF-MS raw data were imported into the software Progenesis QI (Waters Corporation, Milford, MA, USA) for baseline filtering, peak identification, integration, retention time correction and peak alignment, and finally, a data matrix of retention time, mass-to-charge ratio and peak intensity was obtained for subsequent multivariate statistical analysis. Unsupervised principal component analysis (PCA) and orthogonal partial least squares discrimination analysis (OPLS-DA) were performed by R packages (Version 1.6.2). In OPLS-DA analysis, a combination of variable importance in projection (VIP) >1 and *p* value < 0.05 was used to screen out the differential metabolites among different groups. The VIP value can be used to filter the variables that contribute most to the model, and variables with VIP > 1 are often selected as differential metabolites or potential markers. In our study, metabolites were screened based on VIP > 1, *p* < 0.05 and fold change (FC) >1 or FC < 1. Student’s t test was applied to calculate the significance of the metabolite intensity differences among different groups. The heatmap was constructed by Python (Version 1.0.0) and Euclidean was adopted to calculate the metabolite distance. To identify potential markers and metabolic pathways, biochemical databases including the Human Metabolome Database (HMDB) (http://www.hmdb.ca/) and the Kyoto Encyclopedia of Genes and Genomes (KEGG) (http://www.kegg.com/) were applied.

### 2.6. Data Analysis

The data were expressed as mean ± standard error of mean (SEM). Statistical calculations and analyses were performed using SPSS 22.0 software (IBM Corporation, Chicago, IL, USA). One-way analysis of variance (ANOVA) followed by Duncan’s post-hoc test or Student’s t test was adopted for comparisons of group differences. Data with *p* < 0.05 were considered to be statistically significant in this study. 

## 3. Results

### 3.1. Effect of PCFM on Diabetes-Related Biomarkers in Diabetic Mice

**Figure 2:**
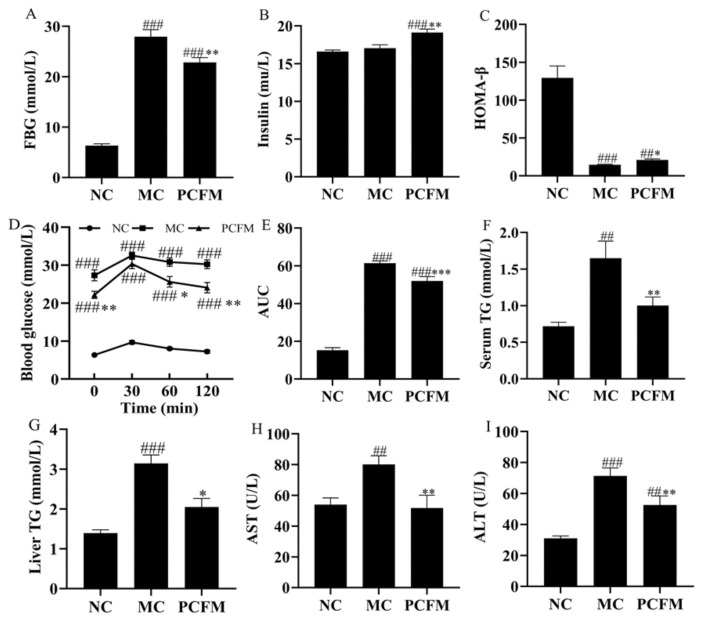
After 5 weeks’ treatment, effect of prolamin from cooked foxtail millet (PCFM) administration on levels of (**A**) Fasting blood glucose (FBG), (**B**) Insulin, (**C**) Homeostasis model assessment—islet β cell function (HOMA-β), (**D**) Oral glucose tolerance test (OGTT), (**E**) Area under curve (AUC), (**F**) Serum triglyceride (TG), (**G**) liver TG, (**H**) Aspartate aminotransferase (AST) and (**I**) Alanine aminotransferase (ALT) in mice. Values were analyzed by One-way analysis of variance (ANOVA) followed by Duncan’s post-hoc test and expressed as the mean ± SEM of 8 mice/group. ### *p* < 0.001, ## *p* < 0.01, compared to normal control (NC) group, *** *p* < 0.001, ** *p* < 0.01, * *p* < 0.05, compared to model control (MC) group.

In contrast to the steady increased BW in the NC group, the mice in the MC group exhibited a significant BW loss (Figure S2), which is a typical symptom of diabetes [30]. After 5 weeks’ treatment with PCFM, the BW of mice was slightly increased, rather than decreased like the mice in the MC group, which suggested the beneficial effect of PCFM supplementation on inhibiting BW loss in diabetic mice, though a significant difference in BW gain was still observed between NC and PCFM groups (Figure S2). In addition, diabetes induced by a high-fat diet combined with low-dosed STZ (90–100 mg/kg) manifested as hyperglycemia, hyperlipidemia, pancreatic β-cell failure, and insulin resistance in mice [36]. The PCFM group showed a significantly lower FBG level compared to the MC group, though the FBG level in the PCFM group was still significantly higher than that in the NC group, (Figure 2A). HOMA-β is an index for assessing the islet β-cell function [33]. Significantly high HOMA-β in the PCFM group demonstrated that PCFM administration ameliorated islet β-cell impairment in diabetic mice, and thus stimulated insulin secretion (Figure 2C), which was confirmed by the significantly higher insulin level in the PCFM group than that in the MC group (Figure 2B). Besides, HOMA-IR is an indicator used to evaluate insulin resistance level [33]. As shown in Figure S3, the HOMA-IR in the MC group was significantly higher than that in the NC group, which suggested the insulin resistance occurrence in diabetic mice. After 5 weeks’ treatment by PCFM, HOMA-IR was decreased compared to that in the MC group (*p* < 0.1), though HOMA-IR in the PCFM group was still significantly higher than that in the NC group. This indicated that PCFM could have a beneficial effect on alleviating insulin resistance in diabetic mice. In addition, the OGTT result proved that the PCFM group exhibited improved glucose intolerance, as evidenced by the significantly lower AUC compared with the MC group (Figure 2D,E). Moreover, the serum and liver TG levels in the MC group were significantly higher than those in the NC group, which corresponded to lipid disorders in diabetic mice. The decreased serum and liver TG levels in PCFM-treated mice indicated that PCFM ameliorated hyperlipidemia in diabetic mice (Figure 2F,G). Furthermore, significantly high AST and ALT levels in diabetic mice suggested liver function impairment accompanied with damaged hepatocyte membrane permeability, which led to the increased release of AST and ALT from hepatic cells into the blood (Figure 2H,I) [37]. PCFM supplementation decreased AST and ALT levels (*p* < 0.01) (Figure 2H,I). This could be because PCFM treatment may alleviate the hepatic cell membrane damage and thus contribute to transmembrane exchange and receptor function in hepatic cells, including the transport of AST and ALT from the blood to hepatocytes. Notably, ALT mainly exists in the liver, while AST is distributed in cardiomyocytes, liver cells and skeletal muscle cells. Therefore, ALT is a much more specific marker-targeted liver function impairment compared to AST [38]. In our study, the amelioration effect of PCFM on liver function impairment in diabetic mice was limited, thus resulting in a significantly higher ALT level than that in the NC group. The lack of significant difference in the AST level between the NC and PCFM groups could be caused by its relatively low specificity towards liver function impairment. The decreased AST and ALT in PCFM-treated mice indicated an improved liver function, and thus contributed to the modulation of glucose homeostasis in diabetic mice.

### 3.2. Effect of Oral PCFM on Gut Microbiota Composition of Mice

**Figure 3:**
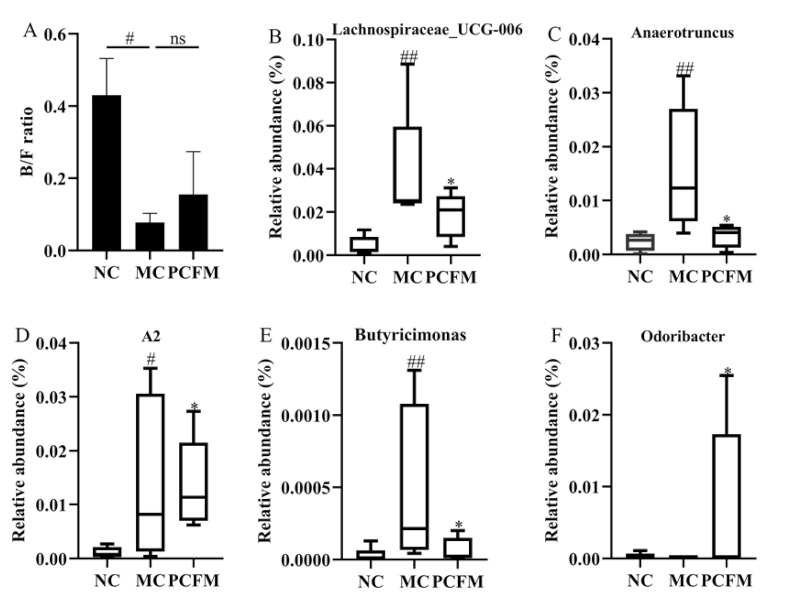
After 5 weeks’ treatment, effect of prolamin from cooked foxtail millet (PCFM) supplementation on the Bacteroides/Firmicutes (B/F) ratio (**A**) and the relative abundance of bacteria at the genus level (**B**–**F**) in all groups (*n* = 7 mice/group). The B/F ratio between two groups was analyzed by Student’s t test. Relative abundance of each bacterium referred to the percent of a specific bacterium abundance in comparison to the sum of all bacteria abundance at the same classification level. Wilcoxon rank sum test was used to compare the significance of relative abundance differences among all groups. # *p* < 0.05, ## *p* < 0.01, compared to the normal control (NC) group, * *p* < 0.05, compared to the model control (MC) group. ns: not significant.

With the hypothesis that PCFM may cause alterations in gut microbiota, the effect of PCFM on the gut microbiota composition of diabetic mice was investigated. Both PCoA and NMDS plots suggested that the gut microbiota composition in diabetic mice was altered evidently after PCFM supplementation, though a small overlap can be observed between the group ellipses of the MC and PCFM groups in the PCoA plot (Figure S4A,B). Firmicutes and bacteroides are two major phyla in the gut microbiota of mice. The ratio of B/F (Bacteroides to Firmicutes) has been reported to be positively related to plasma glucose level [39]. As shown in Figure 3A, the MC group exhibited a significantly lower B/F ratio compared to the NC group, which corresponded to its high FBG level (Figure 2A). Notably, this result was different from that of the above study. PCFM supplementation increased the B/F ratio slightly in diabetic mice, though no significant difference was observed between the MC and PCFM groups (*p* = 0.62). Overall, PCFM administration had no obvious effect on gut microbiota at the phylum level. At the genus level, the relative abundances of *Lachnospiraceae_UCG-006*, *Anaerotruncus*, *A2* and *Butyricimonas* in the MC group were significantly increased when compared with the NC group (*p* < 0.05). PCFM treatment decreased the relative abundances of *Lachnospiraceae_UCG-006*, *Anaerotruncus*, *A2* and *Butyricimonas* (*p* < 0.05) (Figure 3B–E). In addition, the relative abundance of *Odoribacter* was increased in the PCFM group compared with the MC group (*p* < 0.05) (Figure 3F). Collectively, the diabetes-induced relative abundance changes of the above bacteria were altered by PCFM, which may be associated with the anti-diabetic effect of PCFM. 

**Figure 4:**
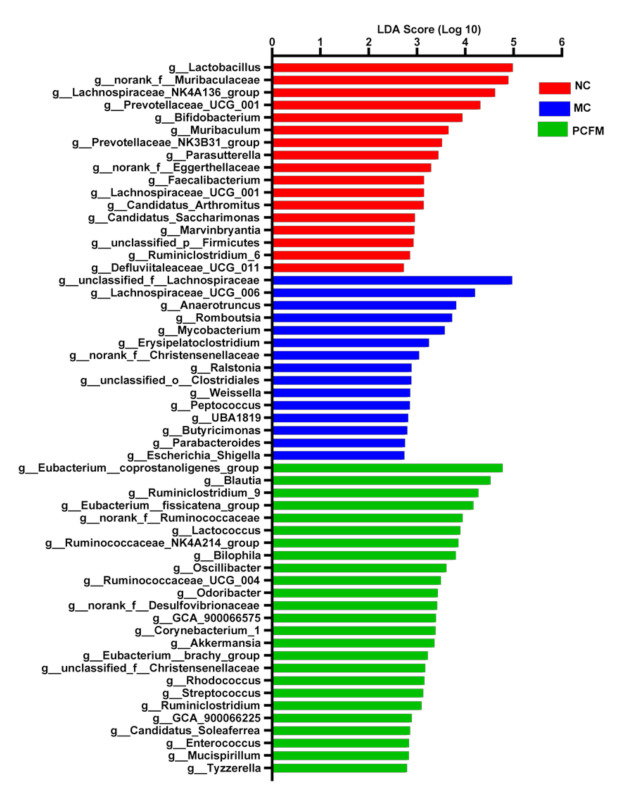
Linear discriminant analysis (LDA) score shows the genus LDA score of > 3 (the length of the bar represents the LDA score) (*n* = 7 mice/group). MC: model control group; NC: normal control group; PCFM: prolamin from cooked foxtail millet group.

LDA score analysis showed that the specific bacterial taxa were different for each group (Figure 4). The NC group was characterized by genera *Lactobacillus*, *norank_f__Muribaculaceae*, *Lachnospiraceae_NK4A136_group* and *Prevotellaceae_UCG_001*. However, the MC group was enriched in *unclassified_f__Lachnospiraceae*, *Lachnospiraceae_UCG-006*, *Anaerotruncus* and *Butyricimonas*, which corresponded to the significantly high relative abundances of *Lachnospiraceae_UCG-006*, *Anaerotruncus* and *Butyricimonas* in the MC group (Figure 3B,C,E). Compared with the NC and MC groups, *Blautia*, *Akkermansia* and *Odoribacter* were enriched in the PCFM group, which was consistent with the significantly high relative abundance of *Odoribacter* in the PCFM group (Figure 3F). Taken together, gut microbiota composition in diabetic mice can be affected by PCFM supplementation. 

In addition, 16S rRNA data were analyzed using the phylogenetic investigation of communities via the reconstruction of unobserved states (PICRUSt) to investigate the underlying pathways that altered gut microbiota in groups. As shown in Figure S5A, 26 KEGG pathways were significantly upregulated, while 50 KEGG pathways were significantly downregulated, in the MC group compared to the NC group. After 5 weeks’ treatment by PCFM, 17 KEGG pathways were upregulated while 13 KEGG pathways were downregulated (*p* < 0.05) (Figure S5B). Notably, our results showed that starch and sucrose metabolism was activated, while peroxisome proliferator-activated receptors (PPARs) signaling pathways were inhibited due to diabetes, which may result in increased glucose levels and impaired energy balance in diabetic mice. Starch can be synthesized from sucrose, and the above two sugars could produce glucose by hydrolysis, which thus increased glucose level and then induced glucose homeostasis dysbiosis. In addition, PPARs play an important role in glucose and lipid metabolism regulation, which are also closely related to the occurrence and development of diabetes and hyperlipidemia. PCFM administration inhibited starch and sucrose metabolism and activated the PPARs signaling pathway, and thus may slow down the glucose production, promote the energy balance, and then alleviate the glucose homeostasis disorders in diabetic mice. Collectively, these results indicated that the anti-diabetic effect of PCFM supplementation was associated with the alteration of gut microbiota composition in diabetic mice.

### 3.3. Serum Metabolites Profiling by UPLC-Q-TOF/MS

**Figure 5:**
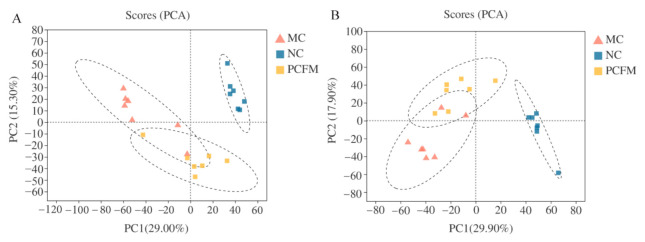
After 5 weeks’ treatment, effects of oral prolamin from cooked foxtail millet (PCFM) on serum metabolic profile in mice. A–B: principal component analysis (PCA) score plots of serum metabolic profiles in all groups ((**A**): positive ion; (**B**): negative ion) (*n* = 7 mice/group). MC: model control group; NC: normal control group; PC1, PC2: principal component 1 and principal component 2.

We further performed metabolomics analysis to investigate the effect of PCFM on serum metabolites in diabetic mice. A PCA score plot was depicted to reflect the distribution of all samples and the dispersion degree among different groups, and the results are presented in Figure 5A,B. PC1 and PC2 scores are the principal components in the PCA model that reflect the main variation and separation in the data. In our study, PC1 and PC2 accounted for 29.0% and 15.3% under a positive model, respectively, and accounted for 29.9% and 17.9% under a negative model, respectively. All the points in the NC group were clearly separated from the MC group, thus indicating the occurrence of metabolic disorders in diabetic mice. The group ellipse of the PCFM group partly coincided with that of the MC group, which also tended to approach the normal group, suggesting the beneficial effect of PCFM on T2DM.

**Figure 6:**
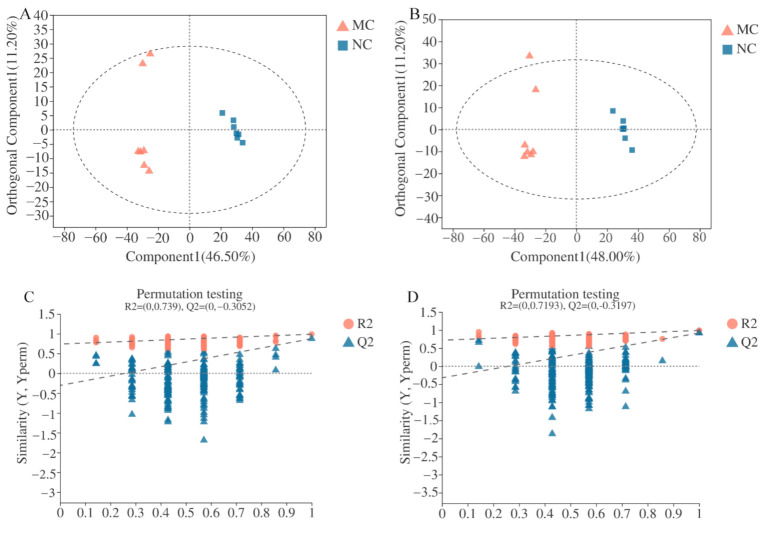
(**A**,**B**): Orthogonal partial least squares discrimination analysis (OPLS-DA) score plots of serum metabolic profiling in normal control (NC) vs. model control (MC) groups under positive-ion (**A**) and negative-ion (**B**) modes (*n* = 7 mice/group). (**C**,**D**): Permutation test in positive- (**C**) and negative (**D**)-ion modes of the NC vs. MC groups. The abscissa represents the replacement retention of the replacement test. The ordinate indicates the value of R2 (red dots) and Q2 (blue triangle) in the substitution tests. The two dashed lines represent the regression lines of R2 and Q2.

In addition, OPLS-DA was carried out to distinguish differential metabolites among different experimental groups. As shown in Figure 6A,B, clusters of MC group were evidently separated from the NC group in both the positive-ion and negative-ion modes. The R2 (evaluating the modeling ability) and Q2 (describing the predictive ability) of the model for the prediction were 0.988 and 0.875 in the positive-ion mode, and 0.99 and 0.92 in negative-ion mode, respectively, indicating that the OPLS-DA models were reliable. Permutation tests were conducted to verify the model. The R2 and Q2 intercepts were 0.739 and 0.3052 in the positive-ion mode, respectively, and 0.7193 and 0.3197 in the negative-ion mode, respectively (Figure 6C,D). The Q2 regression line had a negative intercept, thus demonstrating that the model was not overfitting. Overall, the OPLS-DA score plots demonstrated that the metabolic profile in normal mice was disturbed by diabetes. 

A total of 269 metabolites were annotated and selected as potential biomarkers (VIP > 1 and *p* < 0.05) between the NC and MC groups under two ESI modes (Table S1). Among these metabolites, 28 metabolites were involved in about 24 KEGG pathways, including starch and sucrose metabolism, glycerophospholipid metabolism, and tryptophan metabolism (Table 1, Table S2). Pathway enrichment and topology analysis was applied to explore the possible targeted KEGG pathways influenced by diabetes based on the pathway impact values and *p* values (Figure S6). Generally, pathways with impact value threshold above 0.1 were regarded as potential targeted pathways, and a smaller *p* value indicates more significant enrichment [40]. Two pathways were identified with an impact value above 0.1, which were starch and sucrose metabolism and retinol metabolism (Table S2). The *p* value of starch and sucrose metabolism was lower than 0.5, indicating that the starch and sucrose metabolism may be the potential target pathway that responded to diabetes the most in the present study. Notably, all the 269 metabolites annotated and disturbed by diabetes between the NC and MC groups were matched in the PCFM group. A heatmap was depicted to perform hierarchical clustering analysis of the annotated metabolites, and the results presented the variation trends of these differentially expressed metabolites among the NC, MC and PCFM groups (Figure S7). Metabolites were classified into 10 clusters, and cluster analysis suggested that the serum metabolic profile of the PCFM group was close to that of the NC group, thus indicating that PCFM effectively alleviated diabetes-induced metabolic abnormalities in serum, which contributed to the anti-diabetic effect of PCFM. Among the 269 metabolites matched in the PCFM group, 143 metabolites’ levels were altered after PCFM treatment (*p* < 0.05), and among these, 15 metabolites were involved in KEGG pathways, including serotonin, D-Glucose and so on (Table 1). A further comparison of the NC and PCFM groups indicated that eleven of the above fifteen metabolites were almost restored to a normal level, as no significant difference in these metabolite levels was observed between the NC and PCFM groups (Table 1). Taken together, metabolic disorders induced by diabetes were alleviated by PCFM treatment, which is consistent with the results of PCA plots and indicates the beneficial effect of PCFM in diabetes (Figure 5).

## 4. Discussion

Previous studies have demonstrated the beneficial effects of millet protein on obesity and diabetes. Prolamin in millet protein may be the potential component protein for its anti-diabetic effect [10]. However, the mechanisms associated with the anti-diabetic effect of millet protein remain unclear. In addition, millet is usually consumed after cooking, which process may affect the functions of millet proteins [16,17,18]. As such, we chose foxtail millet as the subject and the hypoglycemic effect of PCFM was explored. In addition, we did not set up the group of mice fed with SCD to be treated with PCFM as well. According to the previous studies concerning the hypoglycemic effect of food proteins or food protein-derived peptides, we found that compared to mice who were only fed with SCD, food proteins or peptides supplementation, including soy protein concentrates, rice bran protein hydrolysates and peptides from potatoes, exhibited no negative effects and induced no significant changes in the biomarkers related to diabetes or obesity in mice fed with SCD [41,42,43,44,45]. Therefore, we considered that prolamin, a major storage protein in millet, could have no obvious effect on the mice fed with SCD. Moreover, physiological saline or PCFM was administrated to mice by gavage for 5 weeks in our study. It is worth noting that mice in the NC group exhibited a normal physiological state, as evidenced by the normal level of FBG and steadily increased BW. Besides, previous studies also support the treatment approach in our study as reasonable. For example, feruloylated oligosaccharides were administrated into type 2 diabetic rats by gavage one per day for 8 weeks, to investigate their anti-diabetic effect [46]. Besides, chitosan oligosaccharides were orally injected into diabetic mice by gavage once per day for 3 months to investigate their effect on T2DM [47]. In addition, flavone hispidulin was supplemented into diabetic mice by oral gavage once per day for 6 weeks to evaluate its anti-diabetic potential [48]. Thus a daily gavage for 5 weeks caused no stress or invasion for mice in the present study. Overall, our study demonstrated that PCFM exerted an anti-diabetic effect by mitigating glucose homeostasis, ameliorating TG accumulation in the serum and liver, alleviating the impairment of pancreatic β-cell function and improving liver function in diabetic mice. In addition, prolamin could produce peptides and amino acids through digestion or gut microbiota proteolysis in vivo; at the same time, absorbed amino acids or short peptides can induce serum metabolites changes [20,21,22]. Therefore, links of PCFM function and gut microbiota along with serum metabolic profile were investigated in this study.

Analysis of the fecal microbiota demonstrated that PCFM attenuated gut microbiota dysbiosis induced by diabetes. Among these genera altered by PCFM supplementation, Lachnospiraceae_UCG-006 was found to be enriched in HFD-fed mice [49], which was consistent with the significantly high relative abundance of *Lachnospiraceae_UCG-006* in the MC group of our study. *Lachnospiraceae_UCG-006* was also reported to be positively correlated with lipid-related indicators (i.e., TG, total cholesterol (TC), high-density liptein cholesterol (HDL-C) and low-density lipoprotein (LDL-C)) [49]. Therefore, the beneficial effect of PCFM administration on serum and liver TG accumulation reduction may be associated with the decreased relative abundance of the *Lachnospiraceae_UCG-006* in PCFM group. Besides, a positive correlation between the abundance of genus *Anaerotruncus* and glucose intolerance was observed in high fat-fed mice [50]. In addition, *Anaerotruncus* was positively associated with FBG levels in diabetic male rats treated with liraglutide, a kind of glucagon-like peptide 1 (GLP-1) adopted to treat T2DM [51]. In our study, the relative abundance of *Anaerotruncus* was significantly higher in the MC group compared to the NC group, while PCFM supplementation significantly decreased the relative abundance of *Anaerotruncus*, and thus may contribute to the glucose-lowering effect and glucose intolerance improvement effects on diabetic mice by PCFM. Moreover, *Butyricimonas* was reported to be positively related to FBG in T2DM patients [52]. The downregulation of *Butyricimonas* levels was also observed in male rats with hyperuricaemia when treated with benzbromarone, a urate-reducing drug used to treat hyperuricaemia [53]. Consistently, a decreased relative abundance of *Butyricimonas* accompanied by a significantly low FBG level was observed in diabetic mice after 5 weeks’ treatment with PCFM. Furthermore, *Odoribacter* was negatively related to steady-state plasma glucose (SSPG) in pre-diabetic individuals, and decreased *Odoribacter* levels were present in insulin-resistant individuals [54,55]. In our case, *Odoribacter* abundance was increased by PCFM. Therefore, the relative abundance changes of the above genera by PCFM treatment could have beneficial effects on the alleviation of T2DM in diabetic mice. Notably, *Blautia* and *Akkermansia* were also enriched in the PCFM group. An increased *Blautia* level was observed in T2DM patients when treated with metformin and berberine, two drugs used for T2DM treatment clinically, which may contribute to the alleviation of inflammation and insulin resistance in diabetes via producing short-chain fatty acids (SCFA), a kind of metabolite proven to have a beneficial effect on glucose metabolism regulation [56]. Besides this, a clinical trial concerning the effect of a herbal formula consisting of eight herbs (AMC), a formula applied in clinical treatment for T2DM patients that has exhibited effectiveness, on patients with T2DM also revealed that the increased *Blautia* abundance by AMC supplementation was positively related to glucose and lipid homeostasis [57]. Moreover, oral *Akkermansia muciniphila* significantly altered gut microbiota composition and thus ameliorated glucose metabolism disorders in STZ-induced diabetic rats [58]. Increased *Akkermansia* abundance was also observed in diabetic patients when treated with metformin [59]. The above two species (*Blautia* and *Akkermansia*) both contributed to the amelioration of glucose homeostasis disorders by PCFM in diabetic mice.

Apart from gut microbiota, the mitigation of serum metabolic profile disorders induced by diabetes was also beneficial to the anti-diabetic effect of PCFM. Among these metabolites influenced by PCFM treatment, D-Glucose participates in starch and sucrose metabolism, glycolysis/ gluconeogenesis and galactose metabolism, and the above pathways are classified into the carbohydrate metabolism. Starch can be synthesized from sucrose by phosphorylation, transfer and synthesis reactions, and the above two sugars both could produce glucose by hydrolysis [60]. Besides this, glucose can be generated from pyruvate and/or lactate via gluconeogenesis, which is then degraded into lactate via glycolysis [61]. In addition, galactose can be oxidized to galactose-1-phosphate, which is then transformed into uridine diphosphogalactose (UDP)-galactose by galactose-1-phosphate uridyltransferase, and finally converted to UDP-glucose by UDP-galactose 4′-epimerase [62,63]. Then, UDP-glucose is phosphorylated to glucose-1-phosphate, and transformed to glucose-6-phosphate to participate in glycolysis and thus produce glucose [64]. Therefore, an activated galactose metabolism may result in increased serum glucose levels, which has been associated with a higher risk of T2DM and can thus contribute to the occurrence of insulin resistance [65]. The upregulation of D-Glucose levels in the MC group suggested the inhibition of glycolysis, and the activation of the gluconeogenesis, starch and sucrose metabolism and the galactose metabolism due to diabetes. A significant reduction in serum glucose level was observed in the PCFM group, which may thus alter the above carbohydrate metabolisms and affect the anti-diabetic effect of PCFM. Besides, serotonin, also called 5-hydroxy tryptamine, is involved in tryptophan metabolism. Serotonin deficiency in the pancreas induced insulin secretion damage in tryptophan hydroxylase-1 deficient (TPH1−/−) mice, as circulating serotonin is responsible for insulin section in pancreatic β cells [66]. In addition, serotonergic drugs, including fenfluramine, fluoxetine and sertraline, improved glucose intolerance and insulin sensitivity in obese and diabetic rats or humans [67]. In the present study, the serotonin level was significantly lower in the MC group compared to NC group, while PCFM administration increased the serotonin level evidently. The upregulation of the serotonin level may improve the function of pancreatic β cells, thus stimulating the insulin secretion and then contributing to the hypoglycemic effect of PCFM, which was consistent with the results of the significantly higher HOMA-β index and insulin levels in the PCFM group compared to the MC group. Moreover, abnormalities in the glycerophospholipid metabolism are one of the features in diabetes [68]. In our study, lysoPC (14:1(9Z)), lysoPC (16:1(9Z)/0:0), lysoPC (22:1(13Z)) and lysoPC (14:1(9Z)) participated in glycerophospholipid metabolism. So far, several types of lysoPCs, including lysoPC (18:1), lysoPC (20:1), and lysoPC (dm16:0), have been reported to be reduced in diabetic rats [69], and lysoPC (dm16:0) was negatively associated with T2DM-related biomarkers, including glycated hemoglobin, ALT, γ-glutamyltransferase, C-reactive protein, TG and HDL [70]. In our cases, lysoPC (22:1(13Z)), lysoPC (14:1(9Z)) and lysoPC (16:1(9Z)/0:0) were reduced in the MC group, while PCFM supplementation increased the levels of the above lysoPCs, thus ameliorating the glycerophospholipid metabolism disorders in diabetic mice. Furthermore, Kwon et al. [71] and Chandramohan et al. [72] have proven the alpha-glucosidase inhibitory activity and anti-inflammatory activity of tyrosol against diabetes, a metabolite involved in tyrosine metabolism. PCFM treatment significantly increased tyrosol levels. Collectively, these results suggest that PCFM supplementation alleviated the serum metabolite aberrations induced by diabetes.

## 5. Conclusions

In our study, the hypoglycemic effect of PCFM on STZ-induced diabetic mice was investigated. The results demonstrated that oral PCFM could ameliorate glucose homeostasis disorders caused by diabetes. In addition, the modulation of gut microbiota composition and serum metabolic disorders contributed to the hypoglycemic effect of PCFM. In summary, our study provides experimental evidence for developing PCFM as a potential food component towards diabetes attenuation, and further studies should be performed to explore the potential peptides targeted for the hypoglycemic effect of PCFM.

## Figures and Tables

**Table 1:** The metabolites that were involved in KEGG pathways.

Metabolite	MC/NC	PCFM/MC	PCFM/NC	KEGG Pathway
Lysophosphatidylcholine (LysoPC) (16:1(9Z)/0:0)	↓ ***	↑ #	↓ &&&	Glycerophospholipid metabolism
Serotonin	↓ ***	↑ ##	↓ &&	Tryptophan metabolism
Uridine diphosphogalactose (UDP)-L-rhamnose	↓ *	↑ #	↓	Amino sugar and nucleotide sugar metabolism
LysoPC (22:1(13Z))	↓ ***	↑ #	↓ &&&	Glycerophospholipid metabolism
LysoPC (14:1(9Z))	↓ **	↑ ###	↑	Glycerophospholipid metabolism
M-Coumaric acid	↓ **	↑ ##	↓	Phenylalanine metabolism
Tyrosol	↓ **	↑ ##	↓	Tyrosine metabolism
Lactosylceramide (d18:1/12:0)	↑ **	↓ #	↑	Sphingolipid metabolism
Prostaglandin J2	↑ ***	↓ ##	↑	Arachidonic acid metabolism
9S,11R,15S-trihydroxy-2,3-dinor-13E-prostaenoic acid-cyclo [8 S,12R]	↑ ***	↓ #	↑	Arachidonic acid metabolism
Estriol	↑ ***	↓ ###	↑	Steroid hormone biosynthesis
9,10,13-Trihydroxyoctadecenoic acid (TriHOME)	↑ ***	↓ ###	↑	Linoleic acid metabolism
9(S)-Hydroperoxyoctadecatrienoic acid (HpOTrE)	↑ ***	↓ ###	↑	alpha-Linolenic acid metabolism
19-Hydroxyandrost-4-ene-3,17-dione	↑ **	↓ ###	↑	Steroid hormone biosynthesis
D-Glucose	↑ ***	↓ #	↑ &&&	Starch and sucrose metabolism; Glycolysis / Gluconeogenesis; Galactose metabolism
LysoPC (20:1(11Z))	↓ ***	↑	↓ &&&	Glycerophospholipid metabolism
LysoPC (20:0/0:0)	↓ ***	↑	↓ &	Glycerophospholipid metabolism
Retinol	↓ ***	↑	↓ &	Retinol metabolism
Glycocholic Acid	↓ ***	↑	↓ &&	Primary bile acid biosynthesis
4-O-alpha-D-Galactopyranuronosyl-D-galacturonic acid	↓ *	↑	↓ &	Pentose and glucuronate interconversions
Sucrose	↑ **	↓	↑ &&	Galactose metabolism; Starch and sucrose metabolism
N6-Acetyl-L-lysine	↑ **	↓	↑	Lysine degradation
5’-Deoxy-5-fluorouridine	↑ **	↓	↑	Xenobiotics biodegradation and metabolism
Thiamine	↑ *	↑	↑	Thiamine metabolism
Acetylcholine	↓ ***	↓	↓ &&&	Bile secretion; Glycerophospholipid metabolism
Phosphoserine	↑ ***	↑	↑ &&&	Glycine, serine and threonine metabolism; Cysteine and methionine metabolism
Phosphatidyl choline (PC) (22:5 (4Z,7Z,10Z,13Z,16Z)/P-18:0)	↑ *	↑ #	↑ &&	Linoleic acid metabolism; Arachidonic acid metabolism; alpha-Linolenic acid metabolism

Note: ↑ and ↓ mean the metabolites up- and downregulated in the model control (MC) vs. normal control (NC) groups and the prolamin from cooked foxtail millet (PCFM) vs. MC groups; * indicates significant difference between NC and MC groups, # indicates significant difference between MC and PCFM groups, & indicates significant difference between NC and PCFM groups. * *p* < 0.05, ** *p* < 0.01, *** *p* < 0.001; # *p* < 0.05, ## *p* < 0.01, ### *p* < 0.001; & *p* < 0.05, && *p* < 0.01, &&& *p* < 0.001. Student’s t test was used to compare the significance of the metabolite level differences among different groups.

## Supplementary Materials

The following are available online at https://www.mdpi.com/2072-6643/12/11/3452/s1, Figure S1: Alpha diversity analysis of samples by rarefaction analysis (A) and Shannon index (B). Figure S2: Effect of PCFM administration on (A) body weight (B) body weight gain in diabetic mice. Figure S3. After 5 weeks’ treatment, effect of PCFM administration on HOMA-IR index in diabetic mice. Figure S4. After 5 weeks’ treatment, effects of PCFM administration on gut microbiota composition in diabetic mice. Figure S5. Predicted metabolic pathways of the fecal microbiome after oral PCFM. Figure S6. Bubble chart showing the key altered metabolic pathways due to diabetes. Figure S7. Heatmap of differential metabolites in all experimental groups. Table S1. List of serum metabolites changed among the NC, MC and PCFM groups. Table S2. Summary of the KEGG pathways influenced by diabetes in mice.
