# Supplementary material for: Hypoglycemic Effect of Prolamin from Cooked Foxtail Millet (Setaria italic) on Streptozotocin-Induced Diabetic Mice
Source: Nutrients. 2020 Nov 11;12(11):3452. doi: 10.3390/nu12113452 (PMC7696583; doi:10.3390/nu12113452)
Supplement: Supplementary file 1 [file nutrients-12-03452-s001.zip › nutrients-971704-supplementary.docx]

Supplementary data

**Hypoglycemic effect of prolamin from cooked foxtail millet on streptozotocin-induced diabetic mice**

**Yongxia Fu**

**Online Supplementary Material**

**Supplemental figures**


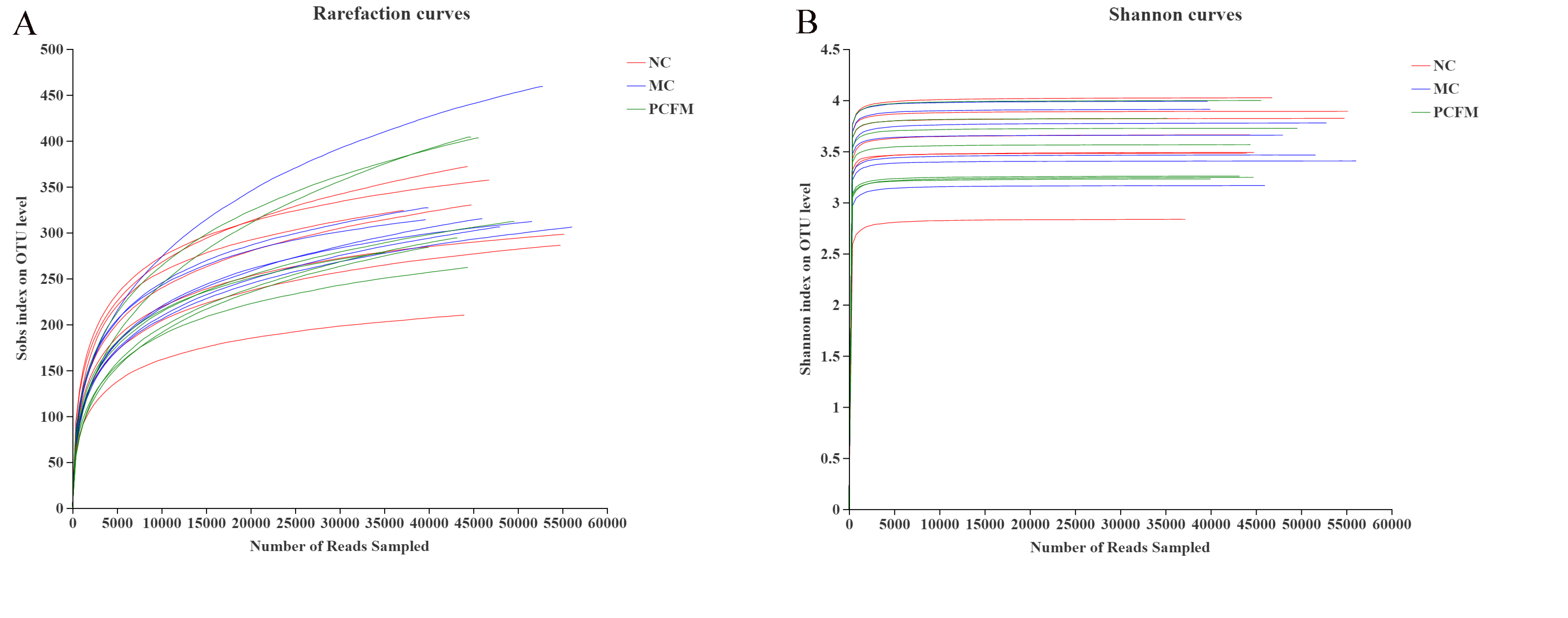


**Figure S1**. Alpha diversity analysis of samples by rarefaction analysis (A) and Shannon index (B) (n=7 mice/group). MC: model control group; NC: normal control group; PCFM: prolamin from cooked foxtail millet group.


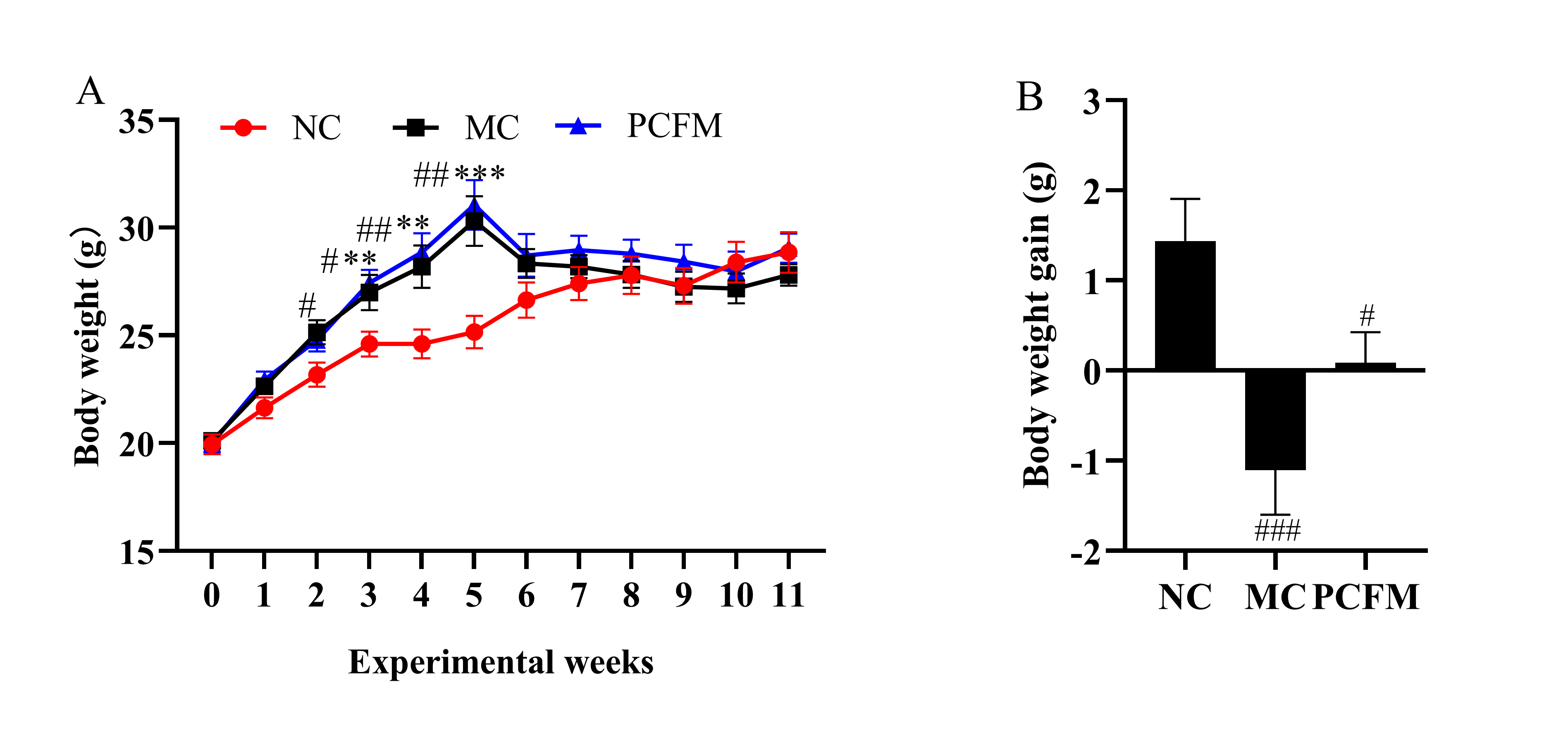


**Figure S2**. After 5 weeks’ treatment, effect of prolamin from cooked foxtail millet (PCFM) administration on (A) body weight (B) body weight gain in diabetic mice. Body weight gain was the differences of body weight in the 7th week and 11th week of the experiment. Values are analyzed by one-way analysis of variance followed by Duncan’s post-hoc test of 8 mice /group. A: #*p* < 0.05, ##*p* < 0.01, model control (MC) vs normal control (NC) groups; ***p* < 0.01, ****p* < 0.001, MC vs PCFM groups. B: #*p* < 0.05, ##*p* < 0.01, compared to NC group.


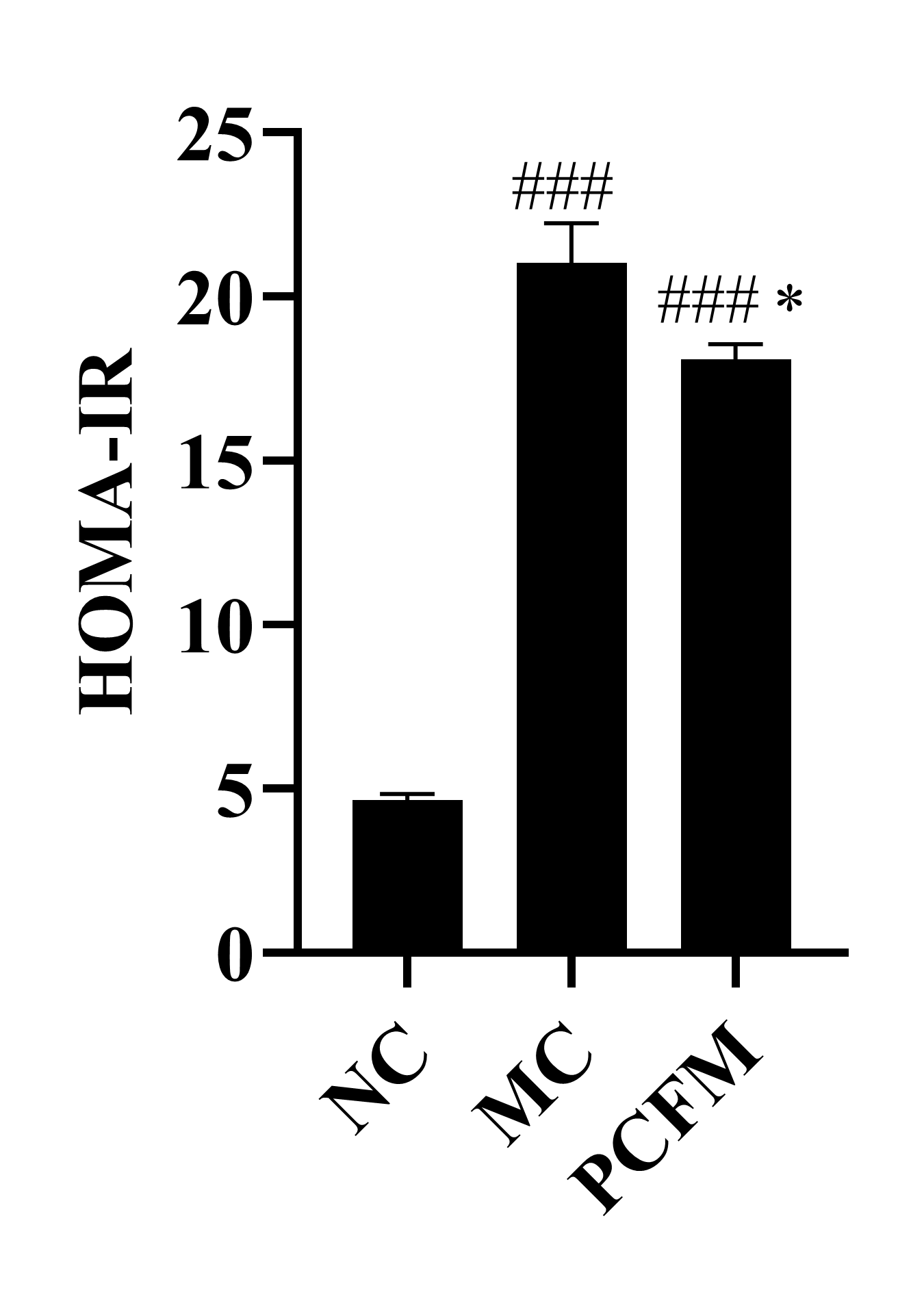


**Figure S3.** After 5 weeks’ treatment, effect of prolamin from cooked foxtail millet (PCFM) administration on homeostasis model assessment-insulin resistance (HOMA-IR) index in diabetic mice. ###*p* < 0.001, compared to normal control (NC); **p* < 0.1, compared to model control (MC) group.


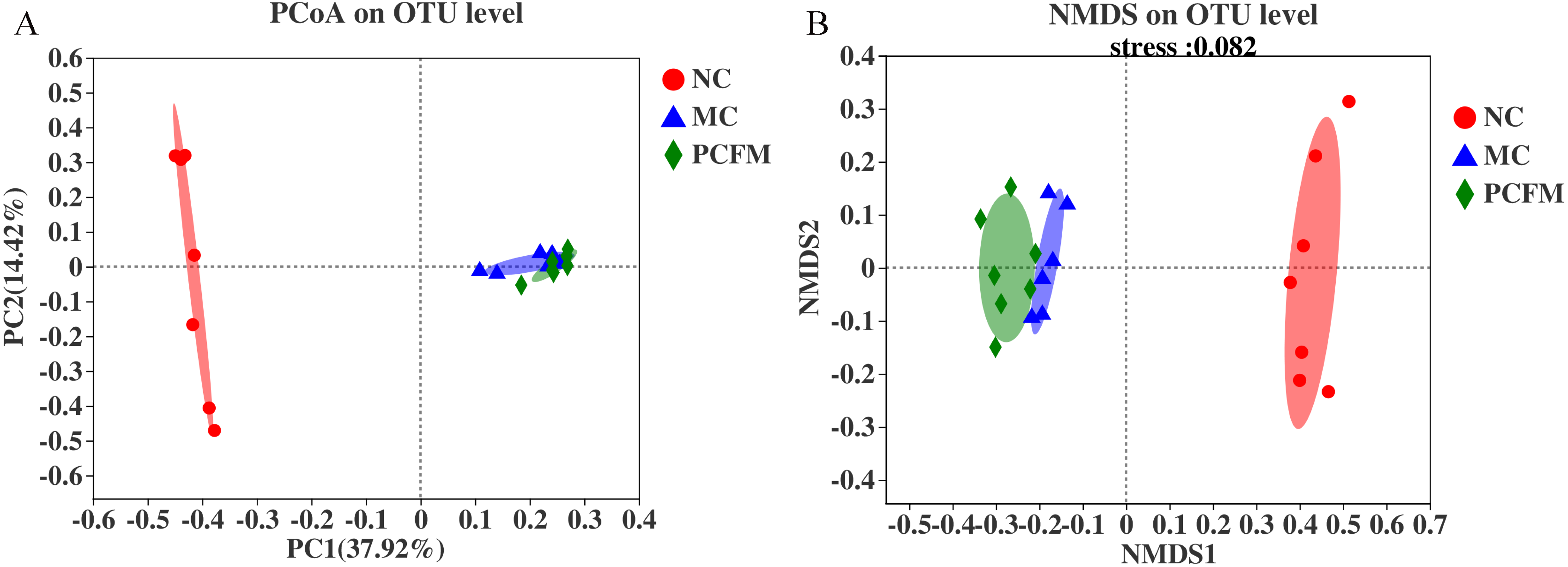


**Figure S4.** After 5 weeks’ treatment, effects of prolamin from cooked foxtail millet (PCFM) administration on gut microbiota composition in diabetic mice (n=6-7 mice/group). A: Principal coordinate analysis (PCoA) score plot; B: Nonmetric multidimensional scaling (NMDS) score plot. PCoA and NMDS plots were analyzed based on Bray–Curtis. NC: normal control group; MC: model control group.


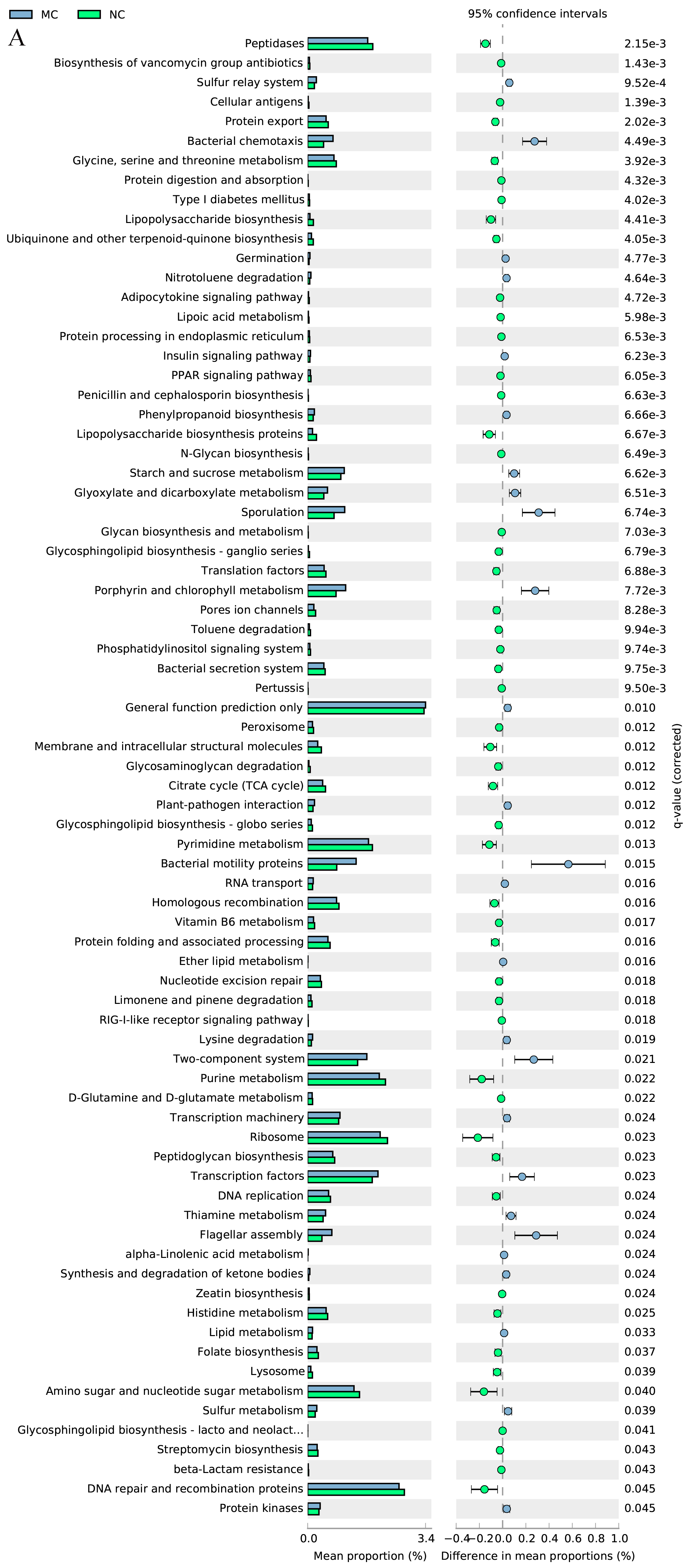


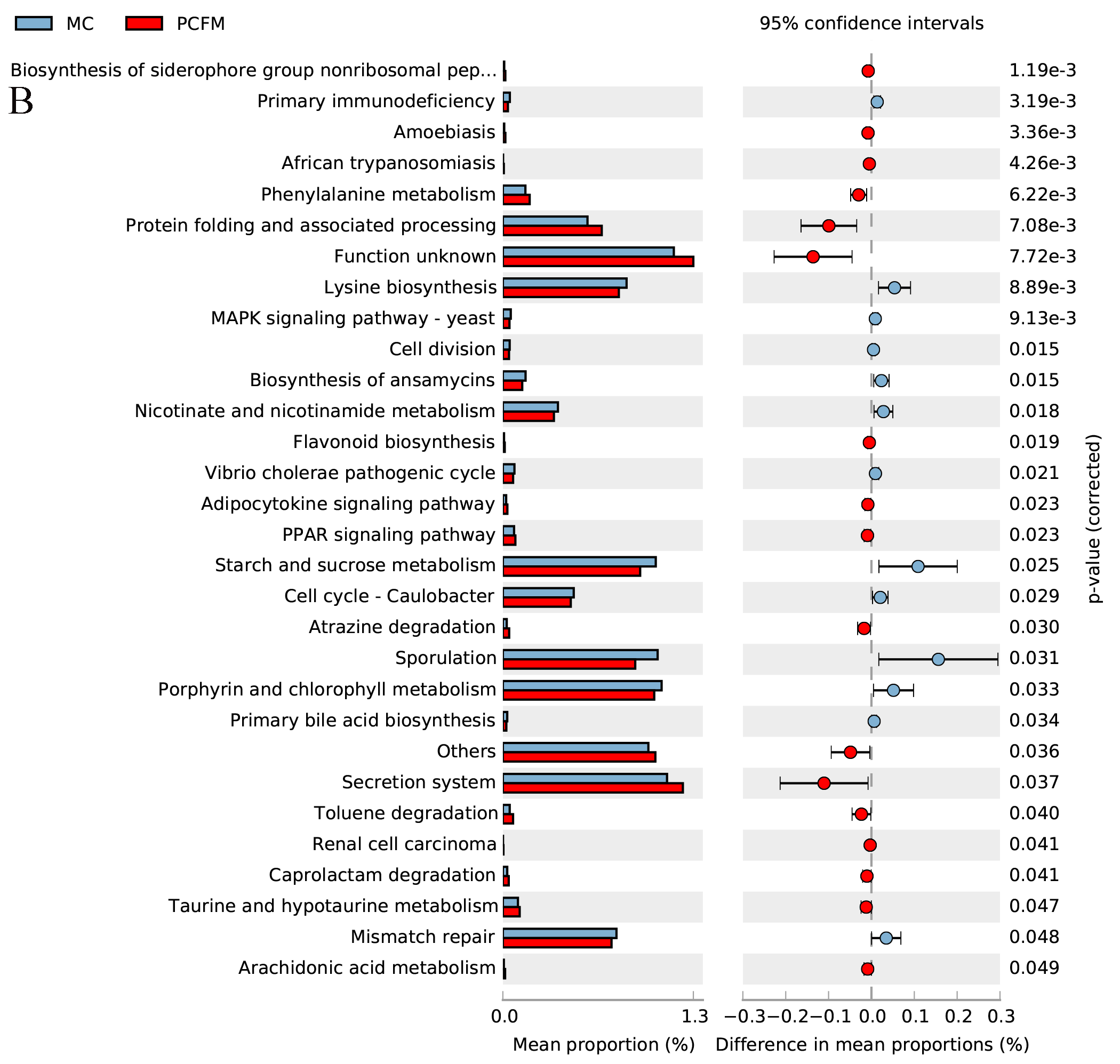


**Figure S5**. Predicted metabolic pathways of the fecal microbiome among all groups (n=7 mice/group). 16S rRNA data were analyzed by the phylogenetic investigation of communities via reconstruction of unobserved states (PICRUSt). Statistical significant difference among all groups was calculated based on Welch’s t test (*p* < 0.05) in STAMP. NC: normal control group; MC: model control group; PCFM: prolamin from cooked foxtail millet group.

**
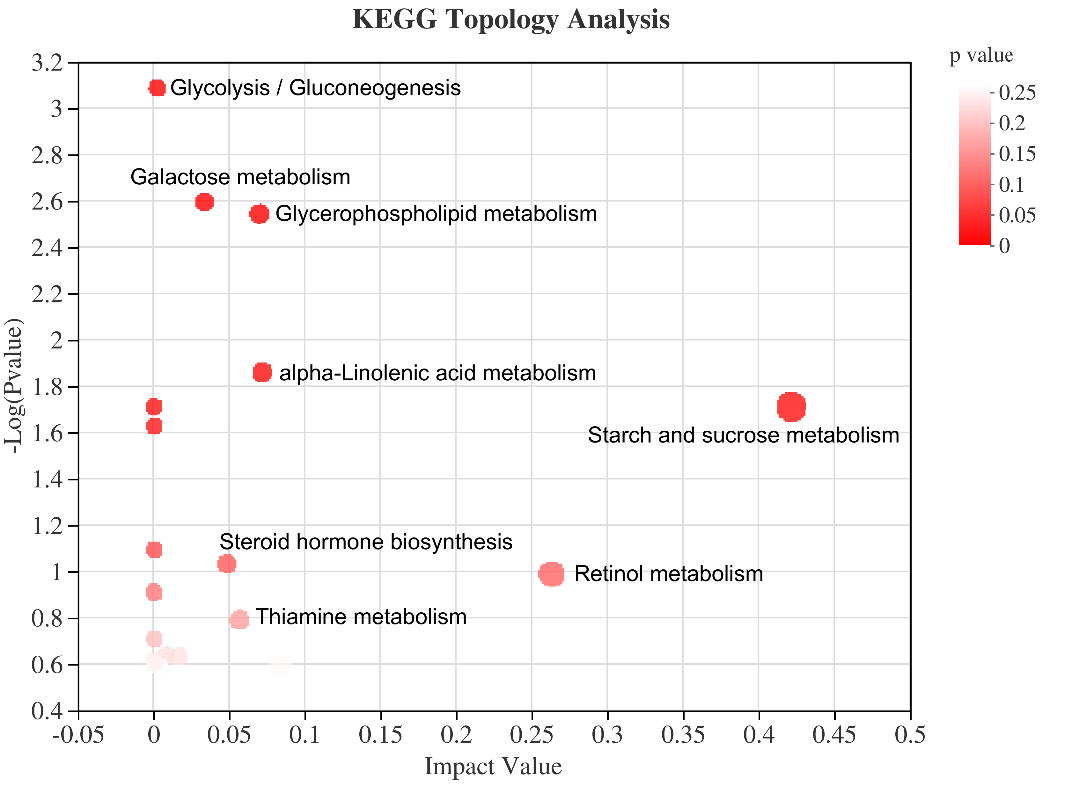
**

**Figure S6.** Bubble chart showing the key altered metabolic pathways due to diabetes. Each bubble in the figure represents a Kyoto Encyclopedia of Genes and Genomes (KEGG) pathway, and the bubble size represents the rich factor: the larger the bubble, the greater the importance.


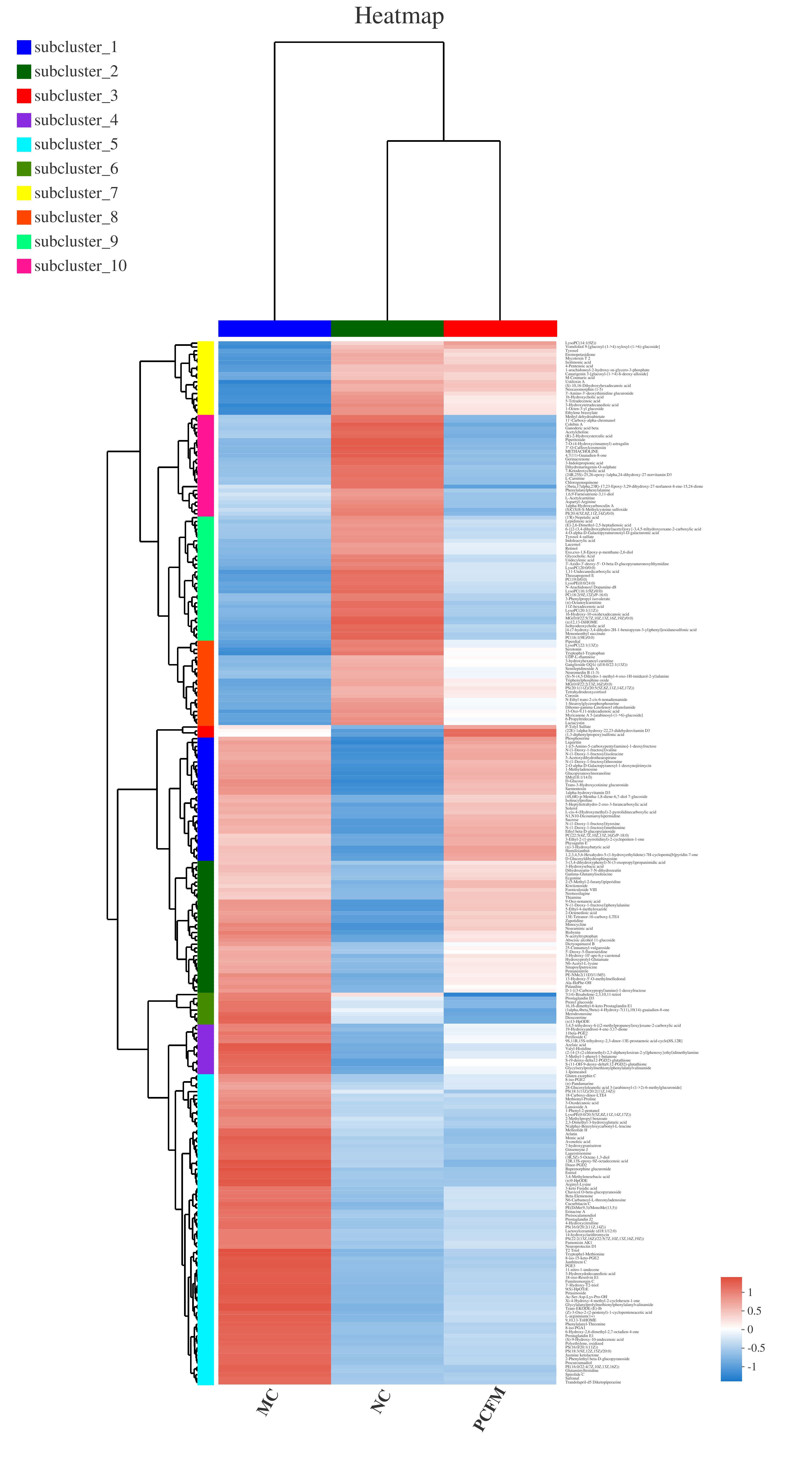


**Figure S7**. Heatmap of differential metabolites in all experimental groups (n=6-7 mice/group). The euclidean was adopted to calculate the metabolite distance. MC: model control group; NC: normal control group; PCFM: prolamin from cooked foxtail millet group. Red represents positive intensity of metabolites while blue represents negative intensity of metabolites.

**Supplementary tables**

**Table S1.** List of serum metabolites changed among NC, MC and PCFM groups

| Metabolite | M/Z | Mode | RT | FC(NC/MC) | P_value | FC(PCFM/MC) | P_value | FC(PCFM/NC) | P_value |
| --- | --- | --- | --- | --- | --- | --- | --- | --- | --- |
| Glycylserylprolylmethionylphenylalanylvalinamide | 634.30 | neg | 5.76 | 0.1735 | 0.000002295 | 0.5017 | 0.001971 | 2.682 | 0.02229 |
| T2 Triol | 383.21 | pos | 5.48 | 0.0623 | 7.106E-09 | 0.1789 | 7.052E-06 | 3.7999 | 0.1441 |
| Buprenorphine glucuronide | 626.33 | pos | 4.62 | 0.1417 | 0.00001323 | 0.1389 | 4.483E-05 | 1.9603 | 0.3454 |
| (2-{4-[3-(2-chloroethyl)-2,3-diphenyloxiran-2-yl]phenoxy}ethyl)dimethylamine | 466.18 | neg | 4.11 | 0.0633 | 0.000002554 | 0.3654 | 0.001595 | 7.3665 | 0.02202 |
| Valyl-Histidine | 550.31 | pos | 6.18 | 0.618 | 2.485E-07 | 0.7194 | 0.0002618 | 1.1951 | 0.06809 |
| 3'-Hydroxy-T2-triol | 381.19 | pos | 5.85 | 0.0357 | 0.00001564 | 0.1805 | 0.0004397 | 7.6945 | 0.08093 |
| Janthitrem C | 606.30 | neg | 5.82 | 0.2033 | 0.00001347 | 0.2766 | 0.0003279 | 1.8993 | 0.179 |
| Petasinoside | 512.22 | neg | 4.23 | 0.1763 | 0.00000371 | 0.2906 | 0.0001068 | 1.6845 | 0.3079 |
| Glycylalanylprolylmethionylphenylalanylvalinamide | 618.30 | neg | 6.21 | 0.1259 | 3.221E-09 | 0.2937 | 9.405E-05 | 2.2547 | 0.1451 |
| S-(9-deoxy-delta12-PGD2)-glutathione | 642.31 | neg | 7.11 | 0.1259 | 1.995E-08 | 0.5301 | 0.002353 | 3.9344 | 0.001072 |
| PS(16:0/20:1(11Z)) | 824.52 | neg | 9.65 | 0.3217 | 0.00002084 | 0.3885 | 0.002278 | 1.4779 | 0.3464 |
| Estriol | 289.18 | pos | 6.83 | 0.2357 | 0.00002184 | 0.2224 | 0.0000498 | 1.0837 | 0.8556 |
| Ac-Ser-Asp-Lys-Pro-OH | 532.23 | neg | 5.15 | 0.114 | 0.00002092 | 0.2658 | 0.0008212 | 2.8544 | 0.1137 |
| Tryptophyl-Methionine | 336.14 | pos | 7.22 | 0.1873 | 1.179E-07 | 0.2833 | 3.712E-05 | 1.9326 | 0.1268 |
| S-(11-OH-9-deoxy-delta9,12-PGD2)-glutathione | 642.31 | neg | 6.24 | 0.2136 | 9.99E-09 | 0.5811 | 0.007094 | 2.6181 | 0.001784 |
| Melleolide H | 413.19 | pos | 5.65 | 0.3757 | 0.0006105 | 0.2819 | 5.811E-05 | 0.9455 | 0.8837 |
| Monic acid | 323.19 | neg | 6.08 | 0.4022 | 0.000004558 | 0.3515 | 0.0001853 | 1.036 | 0.9079 |
| Piperitoside | 575.12 | neg | 5.76 | 3.9226 | 3.688E-12 | 0.9713 | 0.01309 | 0.2473 | 1.45E-15 |
| PE(DiMe(9,3)/MonoMe(13,5)) | 836.55 | neg | 10.03 | 0.2901 | 0.00005702 | 0.4498 | 0.004313 | 1.8087 | 0.1549 |
| Fumitremorgin C | 400.16 | neg | 5.95 | 0.0399 | 4.986E-09 | 0.1653 | 0.0004817 | 7.0599 | 0.2111 |
| Phenylalanyl-Threonine | 308.16 | pos | 4.32 | 0.1656 | 0.00001071 | 0.2425 | 0.0007031 | 2.184 | 0.275 |
| Trans-EKODE-(E)-Ib | 311.22 | pos | 6.23 | 0.5215 | 0.000002937 | 0.6121 | 6.806E-05 | 1.2456 | 0.07418 |
| Ustiloxin A | 694.24 | neg | 6.27 | 5.1774 | 0.00006967 | 4.6499 | 0.000422 | 0.8015 | 0.09972 |
| Tryptophyl-Tryptophan | 389.16 | neg | 5.75 | 3.9793 | 6.65E-08 | 2.5434 | 0.0002182 | 0.6355 | 3.82E-06 |
| PS(16:0/20:2(11Z,14Z)) | 822.51 | neg | 9.65 | 0.3937 | 0.0000464 | 0.4593 | 0.003379 | 1.3562 | 0.3598 |
| 3-Hydroxydodecanedioic acid | 211.13 | pos | 4.87 | 0.3102 | 0.00001109 | 0.4 | 0.0004017 | 1.3609 | 0.3565 |
| Trandolapril-d5 Diketopiperazine | 449.19 | neg | 4.82 | 0.2636 | 0.0002156 | 0.2799 | 0.002013 | 1.4918 | 0.4619 |
| Lactosylceramide (d18:1/12:0) | 850.55 | neg | 9.45 | 0.3452 | 0.002322 | 0.4107 | 0.01939 | 1.473 | 0.3706 |
| 1-Ipomeanol | 169.09 | pos | 4.22 | 0.307 | 0.00002345 | 0.5729 | 0.002095 | 1.6454 | 0.07415 |
| 7-hydroxygranisetron | 349.17 | neg | 5.49 | 0.219 | 0.0002456 | 0.195 | 0.00155 | 1.2339 | 0.7374 |
| Xi-4-Hydroxy-4-methyl-2-cyclohexen-1-one | 171.07 | neg | 3.70 | 0.0831 | 0.000001635 | 0.2273 | 0.001234 | 4.0268 | 0.1521 |
| Azelaic acid | 169.09 | neg | 5.59 | 0.0584 | 0.000000471 | 0.3144 | 0.0003255 | 6.603 | 0.02145 |
| Lagerstroemine | 482.21 | neg | 4.66 | 0.2302 | 0.0008393 | 0.1713 | 0.0009885 | 1.4018 | 0.6745 |
| 18-oxo-Resolvin E1 | 349.20 | pos | 6.12 | 0.6378 | 0.00002433 | 0.6856 | 0.0001061 | 1.1176 | 0.2235 |
| 9S,11R,15S-trihydroxy-2,3-dinor-13E-prostaenoic acid-cyclo[8S,12R] | 329.23 | pos | 6.17 | 0.4657 | 0.0002983 | 0.6903 | 0.02357 | 1.3991 | 0.1677 |
| D-Glucose | 359.12 | neg | 0.70 | 0.3295 | 4.469E-11 | 0.8949 | 0.04478 | 1.336E-10 | 8.29E-09 |
| 11-nitro-1-undecene | 241.19 | pos | 7.06 | 0.2842 | 0.000006282 | 0.3627 | 0.0002272 | 1.3668 | 0.3965 |
| Ethylene brassylate | 307.13 | neg | 6.43 | 4.5437 | 0.00002106 | 3.485 | 0.001438 | 0.6856 | 0.0135 |
| 3-keto Fusidic acid | 594.34 | pos | 6.05 | 0.5129 | 0.0007013 | 0.6067 | 0.002613 | 1.2076 | 0.3831 |
| Theasapogenol E | 503.34 | neg | 7.25 | 2.3221 | 0.000005218 | 1.4566 | 0.01323 | 0.5971 | 3.65E-05 |
| 9(S)-HpOTrE | 309.21 | neg | 7.14 | 0.6581 | 5.155E-07 | 0.7084 | 0.0001596 | 1.1057 | 0.2155 |
| PE-NMe2(11D3/11M5) | 888.57 | pos | 10.17 | 0.5847 | 0.0007808 | 0.8184 | 0.04249 | 1.3317 | 0.04967 |
| 4-Hydroxycitrulline | 572.26 | neg | 5.77 | 0.3009 | 0.000888 | 0.3887 | 0.001929 | 1.6974 | 0.2313 |
| 2-Phenylethyl beta-D-glucopyranoside | 265.11 | neg | 4.73 | 0.2169 | 0.0001117 | 0.314 | 0.0007484 | 1.7737 | 0.3125 |
| PS(22:2(13Z,16Z)/22:5(7Z,10Z,13Z,16Z,19Z)) | 872.57 | pos | 9.73 | 0.6473 | 0.001245 | 0.6869 | 0.01227 | 1.1141 | 0.4468 |
| (Z)-3-Oxo-2-(2-pentenyl)-1-cyclopenteneacetic acid | 207.10 | neg | 6.28 | 0.2127 | 0.00001725 | 0.3821 | 0.0007195 | 2.2296 | 0.05115 |
| 12R,13S-epoxy-9Z-octadecenoic acid | 297.24 | pos | 8.22 | 0.6932 | 0.00001032 | 0.6489 | 1.223E-05 | 0.9792 | 0.7947 |
| (S)-9-Hydroxy-10-undecenoic acid | 242.17 | pos | 4.17 | 0.4746 | 0.00001797 | 0.5459 | 0.001122 | 1.2837 | 0.1791 |
| Erinacine A | 477.25 | neg | 6.63 | 0.1503 | 0.001261 | 0.2138 | 0.001412 | 2.1303 | 0.4518 |
| Glutaminylhistidine | 304.10 | neg | 5.21 | 0.1353 | 0.0002361 | 0.1187 | 0.001206 | 1.8971 | 0.4686 |
| 3,4-Methylenesebacic acid | 209.12 | pos | 5.29 | 0.2365 | 0.0001619 | 0.2201 | 0.0003701 | 1.3897 | 0.4068 |
| Arginyl-Lysine | 283.19 | neg | 6.59 | 0.383 | 0.000005478 | 0.3836 | 0.0002137 | 1.2921 | 0.43 |
| L-argininium(1+) | 158.12 | pos | 4.78 | 0.3962 | 0.00006732 | 0.5108 | 2.653E-05 | 1.4048 | 0.08783 |
| (3beta,17alpha,23R)-17,23-Epoxy-3,29-dihydroxy-27-norlanost-8-ene-15,24-dione | 517.32 | neg | 6.91 | 2.2647 | 0.0002676 | 0.3673 | 0.0308 | 0.2012 | 6.07E-07 |
| 6-Hydroxy-2,6-dimethyl-2,7-octadien-4-one | 213.11 | neg | 4.70 | 0.3421 | 0.0000577 | 0.4055 | 0.0005238 | 1.4167 | 0.2548 |
| Perilloside C | 353.14 | neg | 4.30 | 0.3328 | 0.000002104 | 0.6042 | 0.04319 | 1.4659 | 0.2354 |
| Lansioside A | 680.42 | neg | 8.82 | 0.4016 | 0.01285 | 0.4225 | 0.03268 | 1.3321 | 0.5252 |
| 1-Stearoylglycerophosphoserine | 570.28 | pos | 7.16 | 10.553 | 0.002936 | 5.7419 | 0.01608 | 0.5179 | 0.03307 |
| 8-iso-15-keto-PGE2 | 351.22 | pos | 5.93 | 0.7253 | 0.00004251 | 0.7409 | 4.747E-05 | 1.0539 | 0.412 |
| Jasmine ketolactone | 207.10 | neg | 5.43 | 0.1691 | 0.00074 | 0.2498 | 0.003371 | 2.0274 | 0.1747 |
| 13-Oxo-9,11-tridecadienoic acid | 225.15 | pos | 7.57 | 2.0588 | 0.000008987 | 1.6085 | 0.02642 | 0.7538 | 0.03267 |
| PE(16:0/22:4(7Z,10Z,13Z,16Z)) | 831.56 | pos | 9.02 | 0.5812 | 0.0004154 | 0.5801 | 0.001005 | 1.1083 | 0.3493 |
| Bisbynin | 283.15 | pos | 5.29 | 0.4237 | 9.011E-07 | 0.7836 | 0.02108 | 1.9258 | 0.00032 |
| PGE3 | 349.20 | neg | 6.44 | 0.7152 | 0.00001548 | 0.7375 | 2.178E-05 | 1.0628 | 0.3058 |
| Dinor-PGD2 | 325.20 | pos | 6.26 | 0.6958 | 0.00001528 | 0.6468 | 6.636E-05 |  |  |
| Prostaglandin J2 | 667.42 | neg | 7.09 | 0.3852 | 0.0006936 | 0.4634 | 0.006501 | 1.2277 | 0.517 |
| Serotonin | 177.10 | pos | 1.67 | 1.5318 | 0.00003123 | 1.3007 | 0.003914 | 0.8279 | 0.009133 |
| Polyethylene, oxidized | 225.11 | neg | 5.22 | 0.3979 | 0.00006481 | 0.4808 | 0.001177 | 1.2965 | 0.3495 |
| 3'-Amino-3'-deoxythimidine glucuronide | 438.12 | neg | 5.03 | 4.699 | 0.0002535 | 3.7781 | 0.001368 | 0.755 | 0.09535 |
| (3R,5Z)-5-Octene-1,3-diol | 189.11 | neg | 4.98 | 0.5868 | 0.0002451 | 0.5521 | 0.002256 | 1.0617 | 0.7104 |
| Dioscoretine | 283.20 | pos | 4.33 | 0.4152 | 0.0009108 | 0.1923 | 4.057E-05 | 0.737 | 0.441 |
| Fumonisin AK1 | 638.33 | neg | 5.70 | 0.6623 | 0.0003454 | 0.7257 | 0.0156 | 1.1322 | 0.3534 |
| Cucurbitacin C | 595.30 | neg | 6.69 | 0.7128 | 0.00004674 | 0.7819 | 0.002191 | 1.1337 | 0.08851 |
| 14-hydroxyclarithromycin | 762.46 | neg | 8.91 | 0.5241 | 0.001894 | 0.5813 | 0.02216 | 1.2378 | 0.3158 |
| Eremopetasidione | 237.15 | pos | 7.53 | 2.0917 | 0.001944 | 1.8358 | 0.02047 | 0.7936 | 0.07446 |
| 3,4,5-trihydroxy-6-[(2-methylpropanoyl)oxy]oxane-2-carboxylic acid | 297.12 | pos | 3.90 | 0.6117 | 0.0001068 | 0.7564 | 0.03414 | 1.2036 | 0.1952 |
| Isolimonic acid | 527.19 | neg | 7.42 | 2.168 | 0.001668 | 1.9763 | 0.001281 | 0.8749 | 0.299 |
| 3-Hydroxytetradecanedioic acid | 239.16 | pos | 7.81 | 1.3617 | 3.079E-07 | 1.2415 | 0.0004068 | 0.8914 | 0.03076 |
| Merodesmosine | 439.20 | neg | 4.27 | 0.6031 | 0.0007028 | 0.4092 | 0.0002414 | 0.7992 | 0.1971 |
| 9,10,13-TriHOME | 329.23 | neg | 6.09 | 0.7895 | 0.00002294 | 0.8297 | 0.0002298 | 1.0615 | 0.2003 |
| (±)9-HpODE | 311.22 | neg | 7.16 | 0.7988 | 0.00001173 | 0.8005 | 5.757E-05 | 1.0213 | 0.6437 |
| LysoPC(22:1(13Z)) | 600.40 | pos | 9.68 | 1.2374 | 0.00001487 | 1.1037 | 0.03376 | 0.8869 | 0.000949 |
| Spirolide C | 750.46 | neg | 8.69 | 0.5876 | 0.0007669 | 0.6015 | 0.002209 | 1.1314 | 0.2482 |
| Tyrosol | 119.05 | neg | 4.44 | 4.4508 | 0.001516 | 3.9516 | 0.003471 | 0.7628 | 0.2694 |
| Procurcumadiol | 233.15 | pos | 6.26 | 0.5194 | 0.0001057 | 0.5737 | 0.001324 | 1.1242 | 0.4403 |
| Safranal | 195.10 | neg | 5.45 | 0.3617 | 0.0006783 | 0.3941 | 0.0008914 | 1.2775 | 0.4457 |
| PS(18:3(9Z,12Z,15Z)/20:0) | 796.55 | pos | 9.98 | 0.8323 | 0.00007971 | 0.8498 | 0.005034 | 1.0437 | 0.3293 |
| N6-Carbamoyl-L-threonyladenosine | 456.15 | neg | 7.15 | 0.2377 | 0.0002635 | 0.3808 | 0.01619 | 1.7642 | 0.1646 |
| Preisocalamendiol | 203.18 | pos | 7.11 | 0.5681 | 0.0011 | 0.5961 | 0.004172 | 1.1329 | 0.4803 |
| Vomifoliol 9-[glucosyl-(1->4)-xylosyl-(1->6)-glucoside] | 725.29 | neg | 7.13 | 2.0544 | 0.0008294 | 2.1393 | 0.0005761 | 0.967 | 0.7414 |
| 8-iso-PGA1 | 357.21 | neg | 7.10 | 0.7238 | 0.0003276 | 0.7528 | 0.0001336 | 1.0703 | 0.3191 |
| 1b-Hydroxycholic acid | 423.27 | neg | 6.33 | 2.1515 | 0.00001495 | 1.7693 | 0.003497 | 0.8017 | 0.06153 |
| PC(16:1(9E)/0:0) | 494.32 | pos | 7.87 | 1.1388 | 4.615E-07 | 1.0256 | 0.02853 | 0.9032 | 2.26E-08 |
| Triphenylphosphine oxide | 279.09 | pos | 6.02 | 2.044 | 0.008948 | 1.7107 | 0.002386 | 0.8153 | 0.1627 |
| 16,16-dimethyl-6-keto Prostaglandin E1 | 395.24 | neg | 6.70 | 0.5237 | 0.001979 | 0.3524 | 0.0008368 | 0.7796 | 0.431 |
| Lactacystin | 377.13 | pos | 4.88 | 1.7177 | 0.0002889 | 1.3676 | 0.02096 | 0.7738 | 0.01514 |
| Prostaglandin E1 | 375.22 | neg | 6.62 | 0.8166 | 0.0002436 | 0.8461 | 0.0005067 | 1.0419 | 0.2646 |
| 11beta-PGE2 | 351.22 | neg | 6.41 | 0.8007 | 0.0001229 | 0.8728 | 0.007543 | 1.0911 | 0.0847 |
| (S)-10,16-Dihydroxyhexadecanoic acid | 287.22 | neg | 7.25 | 1.7249 | 0.0008883 | 1.6718 | 0.003233 | 0.9268 | 0.1658 |
| LysoPC(14:1(9Z)) | 510.28 | neg | 7.35 | 1.9577 | 0.0036 | 2.232 | 0.0009292 | 1.1132 | 0.06396 |
| Beta-Elemenone | 251.20 | pos | 7.50 | 0.5493 | 0.001029 | 0.6491 | 0.001971 | 1.2208 | 0.2059 |
| 18-Carboxy-dinor-LTE4 | 462.16 | neg | 4.17 | 0.63 | 0.008179 | 0.6486 | 0.03199 | 1.0857 | 0.7062 |
| Prenyl glucoside | 269.10 | neg | 4.20 | 0.4502 | 0.00868 | 0.2581 | 0.002297 | 0.7351 | 0.3526 |
| M-Coumaric acid | 163.04 | neg | 4.44 | 1.9301 | 0.007783 | 1.8257 | 0.01398 | 0.8437 | 0.3439 |
| MG(0:0/22:2(13Z,16Z)/0:0) | 433.33 | pos | 6.38 | 1.7535 | 0.008711 | 1.4836 | 0.001837 | 0.7903 | 0.06014 |
| Canarigenin 3-[glucosyl-(1->4)-6-deoxy-alloside] | 715.31 | neg | 10.06 | 2.0361 | 0.01436 | 1.9384 | 0.03717 | 0.8849 | 0.4957 |
| 2,3-Dimethyl-3-hydroxyglutaric acid | 209.10 | pos | 4.16 | 0.5501 | 0.006093 | 0.4989 | 0.01666 | 0.89 | 0.7085 |
| N(alpha)-Benzyloxycarbonyl-L-leucine | 266.14 | pos | 4.32 | 0.4303 | 0.009348 | 0.3542 | 0.007969 | 0.814 | 0.6693 |
| N-Arachidonoyl Dopamine-d8 | 448.34 | pos | 7.19 | 1.1644 | 0.0000123 | 1.0474 | 0.02253 | 0.8869 | 4.41E-05 |
| LysoPC(16:1(9Z)/0:0) | 538.32 | neg | 7.94 | 1.1434 | 3.805E-07 | 1.0418 | 0.0297 | 0.912 | 2.81E-08 |
| 13-Hydroxy-5'-O-methylmelledonal | 427.18 | pos | 0.84 | 0.6708 | 0.004386 | 0.8693 | 0.01786 | 1.2194 | 0.1262 |
| LysoPE(0:0/20:5(5Z,8Z,11Z,14Z,17Z)) | 532.30 | pos | 7.22 | 0.6253 | 0.01277 | 0.6051 | 0.005287 | 0.9768 | 0.8957 |
| Chlorogenoquinone | 389.03 | neg | 4.71 | 1.537 | 0.0006963 | 0.9837 | 0.01341 | 0.6371 | 2.9E-05 |
| Ginsenoyne J | 291.20 | neg | 7.90 | 0.7354 | 0.0002099 | 0.7163 | 0.002035 | 1.0051 | 0.951 |
| 1,11-Undecanedicarboxylic acid | 243.16 | neg | 6.63 | 1.2407 | 0.000000147 | 1.0884 | 0.001159 | 0.8674 | 3.96E-08 |
| 4-Pentenoic acid | 299.15 | neg | 6.74 | 2.4158 | 0.006782 | 2.4175 | 0.006288 | 0.9039 | 0.5405 |
| PC(19:0/0:0) | 538.39 | pos | 9.36 | 1.0943 | 2.264E-07 | 1.0333 | 0.0216 | 0.94 | 2.32E-05 |
| Mycotoxin T 2 | 376.11 | neg | 5.73 | 1.3104 | 0.001266 | 1.2599 | 0.003594 | 0.9306 | 0.2816 |
| 7(14)-Bisabolene-2,3,10,11-tetrol | 255.20 | pos | 7.18 | 0.7479 | 0.001808 | 0.2183 | 3.778E-06 | 0.407 | 0.001968 |
| 1-Phenyl-2-pentanol | 209.12 | neg | 5.63 | 0.5199 | 0.0053 | 0.5024 | 0.004371 | 1.0472 | 0.8656 |
| (±)13-HpODE | 293.21 | neg | 7.90 | 0.8468 | 0.001189 | 0.7886 | 0.0002187 | 0.9538 | 0.2323 |
| 3-Methyl-1-phenyl-1-butanone | 347.18 | pos | 5.85 | 0.8353 | 0.00003126 | 0.8883 | 0.002976 | 1.0744 | 0.004165 |
| (22E)-1alpha-hydroxy-22,23-didehydrovitamin D3 | 399.33 | pos | 9.48 | 0.7321 | 0.001378 | 1.2698 | 0.0000155 | 1.5599 | 9.25E-05 |
| 19-Hydroxyandrost-4-ene-3,17-dione | 347.18 | neg | 7.36 | 0.7586 | 0.006408 | 0.8385 | 0.0005976 | 1.1546 | 0.08555 |
| Avenoleic acid | 319.23 | pos | 7.86 | 0.8695 | 0.00009612 | 0.8561 | 0.0003206 | 0.9888 | 0.7146 |
| 1-Octen-3-yl glucoside | 255.16 | pos | 6.22 | 1.289 | 0.00003583 | 1.2004 | 0.002287 | 0.9193 | 0.005601 |
| (1alpha,4beta,5beta)-4-Hydroxy-7(11),10(14)-guaiadien-8-one | 267.20 | pos | 7.36 | 0.7158 | 0.001775 | 0.5171 | 0.0004561 | 0.8439 | 0.2934 |
| Prostaglandin D3 | 315.20 | pos | 7.07 | 0.7821 | 0.00135 | 0.6646 | 0.004169 | 0.8923 | 0.2554 |
| Neuroprotectin D1 | 405.23 | neg | 7.40 | 0.7946 | 0.01001 | 0.8276 | 0.0007542 | 1.0254 | 0.7186 |
| PS(18:1(11Z)/20:2(11Z,14Z)) | 858.55 | neg | 9.74 | 0.771 | 0.026 | 0.6813 | 0.0122 | 0.9287 | 0.6428 |
| 2-Methylpropyl benzoate | 401.20 | neg | 6.73 | 0.5944 | 0.007468 | 0.5054 | 0.02091 | 0.7796 | 0.3481 |
| Neocasomorphin (1-5) | 624.26 | neg | 8.28 | 1.3648 | 0.0004911 | 1.3247 | 0.004548 | 0.9814 | 0.5761 |
| (1,3-diphenylpropoxy)sulfonic acid | 291.07 | neg | 6.65 | 0.6174 | 0.001083 | 1.4538 | 0.006316 | 2.1903 | 6.92E-06 |
| Methionyl-Proline | 534.24 | pos | 4.47 | 0.6857 | 0.0135 | 0.6685 | 0.008395 | 0.9772 | 0.8951 |
| Ala-HoPhe-OH | 357.11 | neg | 5.45 | 0.6914 | 0.007411 | 0.8749 | 0.004761 | 1.2197 | 0.08171 |
| PC(22:5(4Z,7Z,10Z,13Z,16Z)/P-18:0) | 864.62 | neg | 11.51 | 0.7299 | 0.02402 | 1.0658 | 0.03688 | 1.4082 | 0.002938 |
| Arlatin | 267.16 | pos | 5.49 | 0.8026 | 0.0006964 | 0.7672 | 0.0001167 | 0.9958 | 0.9519 |
| 3-Oxodecanoic acid | 231.12 | neg | 4.78 | 0.6281 | 0.01157 | 0.6379 | 0.006063 | 1.1087 | 0.5749 |
| 8-iso-PGE2 | 351.22 | neg | 6.20 | 0.8439 | 0.01142 | 0.8801 | 0.02377 | 1.0251 | 0.7066 |
| Chavicol O-beta-glucopyranoside | 297.13 | pos | 6.56 | 0.7183 | 0.003008 | 0.775 | 0.01158 | 1.1398 | 0.1357 |
| 5-Tetradecenoic acid | 271.19 | neg | 7.32 | 1.2996 | 0.000258 | 1.1961 | 0.003311 | 0.8649 | 0.04386 |
| 1-arachidonoyl-2-hydroxy-sn-glycero-3-phosphate | 459.25 | pos | 8.90 | 1.1511 | 0.003702 | 1.1482 | 0.01263 | 0.9925 | 0.8022 |
| (±)12,13-DiHOME | 297.24 | pos | 9.69 | 20.5818 | 1.456E-11 | 3.7692 | 0.0609 | 0.1679 | 8.44E-11 |
| [4-(7-hydroxy-3,4-dihydro-2H-1-benzopyran-3-yl)phenyl]oxidanesulfonic acid | 321.04 | neg | 4.71 | 4.3511 | 0.000002173 | 1.6981 | 0.1452 | 0.3537 | 5.1E-10 |
| MG(0:0/22:5(7Z,10Z,13Z,16Z,19Z)/0:0) | 449.29 | neg | 7.66 | 13.0089 | 7.609E-10 | 2.1951 | 0.3232 | 0.1847 | 2.44E-09 |
| 7-Ketodeoxycholic acid | 389.27 | pos | 6.45 | 3.0724 | 0.0001848 | 1.0115 | 0.9776 | 0.3543 | 8.99E-06 |
| Cohibin A | 593.48 | neg | 9.69 | 1.9418 | 8.419E-10 | 0.823 | 0.08199 | 0.4221 | 5.83E-11 |
| 4,7(11)-Guaiadien-8-one | 251.20 | pos | 9.55 | 16.4665 | 1.175E-08 | 1.4442 | 0.07482 | 0.0792 | 5.97E-11 |
| 7-O-(4-Hydroxycinnamoyl) astragalin | 575.12 | neg | 5.92 | 9.0826 | 7.97E-13 | 1.0852 | 0.4458 | 0.1572 | 3E-11 |
| 3'-Azido-3'-deoxy-5'- O-beta-D-glucopyranuronosylthymidine | 444.13 | pos | 4.92 | 3.143 | 0.0001242 | 1.688 | 0.2363 | 0.5155 | 0.003206 |
| Glycocholic Acid | 466.32 | pos | 6.28 | 4.2528 | 0.0005113 | 1.7954 | 0.237 | 0.4236 | 0.001936 |
| Myricanene A 5-[arabinosyl-(1->6)-glucoside] | 599.25 | pos | 6.02 | 5.4925 | 0.0001021 | 3.4595 | 0.08493 | 0.5927 | 0.02837 |
| Isohyodeoxycholic acid | 393.30 | pos | 8.88 | 1.9514 | 0.000004527 | 1.225 | 0.1113 | 0.5998 | 6.76E-07 |
| 9-Oxo-nonanoic acid | 217.11 | neg | 5.19 | 0.2129 | 8.72E-12 | 0.8399 | 0.3635 | 3.9077 | 0.000591 |
| Ethyl beta-D-glucopyranoside | 229.07 | neg | 1.37 | 0.4878 | 0.00002549 | 1.0028 | 0.9825 | 2.0672 | 0.000119 |
| 13E-Tetranor-16-carboxy-LTE4 | 378.13 | pos | 1.94 | 0.4824 | 0.00002416 | 0.8969 | 0.3774 | 1.8407 | 0.001033 |
| 2-Octenedioic acid | 153.06 | neg | 3.85 | 0.0867 | 2.249E-07 | 0.7855 | 0.4678 | 9.2715 | 0.01113 |
| Monomenthyl succinate | 293.12 | neg | 5.95 | 1.7825 | 9.629E-07 | 1.1245 | 0.1709 | 0.6058 | 6.38E-07 |
| Ganoderic acid beta | 545.31 | neg | 6.90 | 3.2341 | 0.00002809 | 0.7445 | 0.1026 | 0.2235 | 1.22E-07 |
| 5-Ethyl-4-methyloxazole | 156.07 | neg | 4.22 | 0.2312 | 0.00001791 | 0.8644 | 0.3814 | 3.4062 | 0.002612 |
| Glucopyranosylmoranoline | 362.08 | neg | 0.88 | 0.5221 | 0.000000151 | 0.9638 | 0.5421 | 1.862 | 1.98E-08 |
| 3-Ethyl-2-(1-pyrrolidinyl)-2-cyclopenten-1-one | 224.13 | neg | 4.99 | 0.1503 | 0.0009606 | 1.1487 | 0.6788 | 6.6528 | 0.002285 |
| N-(1-Deoxy-1-fructosyl)threonine | 280.10 | neg | 0.75 | 0.3058 | 1.343E-07 | 0.932 | 0.6544 | 3.0721 | 2.57E-05 |
| Isoleucylproline | 211.14 | pos | 3.84 | 0.1922 | 0.001203 | 0.8857 | 0.74 | 4.0305 | 0.01771 |
| 5-Acetoxydihydrotheaespirane | 299.19 | neg | 7.59 | 0.225 | 0.00006087 | 1.0178 | 0.8143 | 4.9493 | 5.99E-06 |
| 1,2,3,4,5,6-Hexahydro-5-(1-hydroxyethylidene)-7H-cyclopenta[b]pyridin-7-one | 212.13 | pos | 4.37 | 0.5714 | 0.0003709 | 1.067 | 0.6085 | 1.751 | 0.000236 |
| 6-{[2-(3,4-dihydroxyphenyl)acetyl]oxy}-3,4,5-trihydroxyoxane-2-carboxylic acid | 309.06 | pos | 4.39 | 2.9458 | 0.004147 | 1.2558 | 0.6988 | 0.4137 | 0.006224 |
| Sarmentosin | 339.11 | pos | 0.59 | 0.5569 | 0.00000213 | 0.9359 | 0.1822 | 1.7088 | 1.55E-06 |
| Physagulin E | 745.28 | pos | 4.12 | 0.1667 | 0.004474 | 1.1393 | 0.6706 | 8.0405 | 0.000168 |
| Trans-3-Hydroxycotinine glucuronide | 407.10 | pos | 0.59 | 0.549 | 7.393E-08 | 0.9224 | 0.1392 | 1.7274 | 5.21E-07 |
| Acetylcholine | 146.12 | pos | 0.74 | 1.8825 | 6.882E-10 | 0.8567 | 0.408 | 0.4919 | 7.98E-06 |
| (±)-3-Hydroxybutyric acid | 103.04 | neg | 0.88 | 0.627 | 0.0000144 | 1.0545 | 0.5744 | 1.6878 | 2.65E-05 |
| Methyl dehydroabietate | 315.23 | pos | 7.85 | 1.5724 | 0.000003712 | 0.9352 | 0.6362 | 0.6204 | 3.33E-05 |
| Sucrose | 365.11 | pos | 0.70 | 0.6086 | 0.001136 | 0.9859 | 0.6801 | 1.5977 | 0.00196 |
| Palaudine | 370.13 | neg | 4.58 | 0.4572 | 0.0002024 | 0.7219 | 0.09656 | 1.5945 | 0.07464 |
| Gamma-Glutamylisoleucine | 225.12 | pos | 3.70 | 0.4515 | 0.0366 | 0.9286 | 0.818 | 1.8326 | 0.04748 |
| Abscisic alcohol 11-glucoside | 393.19 | neg | 6.22 | 0.4559 | 0.0008016 | 0.8135 | 0.07339 | 1.7245 | 0.008836 |
| Neuraminic acid | 309.13 | pos | 0.73 | 0.7342 | 1.853E-07 | 0.9369 | 0.1462 | 1.0713 | 0.1601 |
| LysoPC(20:0/0:0) | 574.39 | pos | 9.68 | 1.1809 | 8.122E-07 | 1.061 | 0.1597 | 0.8923 | 0.01016 |
| N-(1-Deoxy-1-fructosyl)isoleucine | 292.14 | neg | 1.38 | 0.6839 | 0.000001042 | 1.0058 | 0.9141 | 1.4783 | 8.89E-08 |
| PS(20:1(11Z)/20:5(5Z,8Z,11Z,14Z,17Z)) | 858.53 | pos | 7.64 | 1.8641 | 0.001249 | 1.6429 | 0.07682 | 0.8015 | 0.149 |
| 1alpha-hydroxyvitamin D3 | 401.34 | pos | 9.93 | 0.7925 | 0.00001494 | 0.9667 | 0.2791 | 1.2103 | 1.16E-05 |
| 16-Hydroxy-10-oxohexadecanoic acid | 285.21 | neg | 6.63 | 1.889 | 0.00003867 | 1.1199 | 0.4971 | 0.5614 | 4.39E-07 |
| N6-Acetyl-L-lysine | 171.11 | pos | 3.83 | 0.61 | 0.005231 | 0.8627 | 0.3387 | 1.2923 | 0.1804 |
| Pentanenitrile | 125.11 | pos | 3.82 | 0.4415 | 0.006248 | 0.7939 | 0.2709 | 1.4461 | 0.2437 |
| D-Glucosyldihydrosphingosine | 464.36 | pos | 7.25 | 0.3135 | 0.003261 | 1.0948 | 0.6888 | 3.4759 | 1.51E-06 |
| Exo,exo-1,8-Epoxy-p-menthane-2,6-diol | 228.16 | pos | 6.49 | 2.849 | 0.004471 | 1.6709 | 0.3072 | 0.5 | 0.006317 |
| Zapotidine | 166.04 | neg | 5.08 | 0.2921 | 0.0003497 | 0.8262 | 0.219 | 1.9442 | 0.05468 |
| N1,N10-Dicoumaroylspermidine | 472.20 | neg | 1.37 | 0.3264 | 0.0007499 | 1.0066 | 0.9785 | 3.1854 | 9.91E-05 |
| 4-O-alpha-D-Galactopyranuronosyl-D-galacturonic acid | 403.11 | pos | 4.11 | 6.2823 | 0.01123 | 1.8831 | 0.5498 | 0.3725 | 0.0328 |
| 28-Glucosyloleanolic acid 3-[arabinosyl-(1->2)-6-methylglucuronide] | 482.25 | pos | 3.91 | 0.3051 | 0.02385 | 0.4061 | 0.09357 | 1.6416 | 0.5778 |
| Indoleacrylic acid | 186.06 | neg | 5.13 | 3.63 | 0.003056 | 1.7928 | 0.2125 | 0.4761 | 0.01076 |
| Neotussilagine | 244.12 | neg | 4.51 | 0.4272 | 0.004339 | 1.0171 | 0.9575 | 2.2127 | 0.02457 |
| Thiamine | 310.08 | pos | 5.12 | 0.5727 | 0.04014 | 1.0228 | 0.9148 | 1.5788 | 0.05498 |
| Neuromedin B (1-3) | 285.16 | pos | 3.64 | 4.4053 | 0.03128 | 2.4422 | 0.2379 | 0.5216 | 0.12 |
| Kiwiionoside | 448.25 | pos | 7.37 | 0.36 | 0.002598 | 1.1222 | 0.7264 | 3.0587 | 0.0219 |
| Tyrosol 4-sulfate | 217.02 | neg | 4.79 | 2.7479 | 0.0034 | 1.2787 | 0.6045 | 0.4518 | 0.008429 |
| 1-Methyladenosine | 282.12 | pos | 0.80 | 0.7021 | 6.199E-07 | 0.9773 | 0.5867 | 1.3813 | 1.59E-06 |
| 5-Heptyltetrahydro-2-oxo-3-furancarboxylic acid | 209.12 | neg | 5.37 | 0.4939 | 0.00002619 | 0.9498 | 0.7018 | 1.8942 | 0.001151 |
| METHACHOLINE | 160.13 | pos | 0.78 | 1.2272 | 1.452E-07 | 1.0111 | 0.5715 | 0.8263 | 3.59E-08 |
| L-Acetylcarnitine | 407.24 | pos | 0.82 | 2.0248 | 0.01355 | 0.7573 | 0.6084 | 0.3281 | 0.009749 |
| Phosphoserine | 415.01 | neg | 3.71 | 0.4883 | 0.000004857 | 1.047 | 0.4002 | 2.3155 | 4.16E-08 |
| Dihydrozeatin-7-N-dihydrozeatin | 366.18 | pos | 1.86 | 0.4334 | 0.007939 | 0.885 | 0.6885 | 1.8555 | 0.08499 |
| L-Carnitine | 162.11 | pos | 0.71 | 1.2479 | 0.00001277 | 0.9868 | 0.8277 | 0.7827 | 0.000143 |
| N-(1-Deoxy-1-fructosyl)phenylalanine | 328.14 | pos | 1.16 | 0.8083 | 3.892E-09 | 0.964 | 0.3994 | 1.2062 | 0.000076 |
| 11'-Carboxy-alpha-chromanol | 417.30 | neg | 9.25 | 1.3092 | 0.00004918 | 0.9514 | 0.4653 | 0.6915 | 0.000422 |
| (±)-Octanoylcarnitine | 288.22 | pos | 4.89 | 1.2908 | 0.00001607 | 1.0248 | 0.5881 | 0.7742 | 2.48E-06 |
| 3-(3,4-dihydroxyphenyl)-N-(3-oxopropyl)propanimidic acid | 270.13 | pos | 4.74 | 0.3165 | 0.01607 | 0.8395 | 0.7244 | 2.5598 | 0.1673 |
| Undecylenic acid | 229.15 | neg | 5.53 | 1.896 | 0.0001916 | 1.2097 | 0.2859 | 0.6361 | 5.66E-05 |
| Retinol | 269.23 | pos | 9.13 | 1.3188 | 0.0002705 | 1.1183 | 0.3411 | 0.8455 | 0.04777 |
| Ganglioside GQ1c (d18:0/22:1(13Z)) | 833.08 | pos | 4.99 | 1.6506 | 0.02015 | 1.3581 | 0.05096 | 0.7556 | 0.1172 |
| 3-Hydroxy-10'-apo-b,y-carotenal | 373.25 | neg | 9.37 | 0.6499 | 0.01867 | 0.8923 | 0.05522 | 1.2743 | 0.1154 |
| Corosin | 499.30 | neg | 7.84 | 1.6704 | 0.007025 | 1.4516 | 0.06106 | 0.8331 | 0.1298 |
| 5'-Deoxy-5-fluorouridine | 281.03 | neg | 5.07 | 0.6146 | 0.008912 | 0.7929 | 0.3093 | 1.0234 | 0.9302 |
| 3-Phenylpropyl isovalerate | 221.15 | pos | 5.97 | 1.756 | 0.00248 | 1.0941 | 0.3357 | 0.6041 | 0.000524 |
| Humilixanthin | 349.10 | pos | 0.82 | 0.691 | 0.0005685 | 1.0491 | 0.5221 | 1.489 | 0.000265 |
| 1-[(5-Amino-5-carboxypentyl)amino]-1-deoxyfructose | 353.13 | pos | 0.59 | 0.7613 | 1.277E-07 | 0.9907 | 0.8368 | 1.2996 | 6.32E-06 |
| P-Tolyl Sulfate | 187.01 | neg | 4.61 | 0.4097 | 0.0352 | 1.3048 | 0.2653 | 3.6142 | 0.002939 |
| Piperdial | 295.16 | neg | 6.72 | 1.4996 | 0.000243 | 1.2172 | 0.06703 | 0.7685 | 0.000251 |
| (4S,6R)-p-Mentha-1,8-diene-6,7-diol 7-glucoside | 365.13 | neg | 3.80 | 0.7122 | 0.00152 | 0.9488 | 0.6107 | 1.3531 | 0.008464 |
| (S)C(S)S-S-Methylcysteine sulfoxide | 134.03 | pos | 3.95 | 2.3198 | 0.01354 | 0.5633 | 0.1227 | 0.3173 | 0.001005 |
| D-1-[(3-Carboxypropyl)amino]-1-deoxyfructose | 246.10 | neg | 3.90 | 0.5829 | 0.001007 | 0.8158 | 0.2204 | 1.5113 | 0.0175 |
| Dihomo-gamma-Linolenoyl ethanolamide | 332.29 | pos | 9.75 | 1.3657 | 0.003432 | 1.1706 | 0.171 | 0.8349 | 0.0108 |
| Semilepidinoside A | 301.12 | pos | 5.84 | 1.5565 | 0.02084 | 1.2452 | 0.2972 | 0.7863 | 0.05019 |
| SM(d18:1/14:0) | 719.54 | neg | 10.73 | 0.8177 | 0.000001473 | 0.9846 | 0.4115 | 1.2023 | 1.18E-06 |
| L-cis-4-(Hydroxymethyl)-2-pyrrolidinecarboxylic acid | 128.07 | pos | 1.38 | 0.6613 | 0.001002 | 0.9544 | 0.6228 | 1.2535 | 0.0972 |
| UDP-L-rhamnose | 549.06 | neg | 0.85 | 1.6106 | 0.01274 | 1.3634 | 0.041 | 0.797 | 0.121 |
| Tetrahydrodeoxycortisol | 395.24 | neg | 5.23 | 2.1993 | 0.01245 | 1.8397 | 0.02228 | 0.7726 | 0.1888 |
| 25-Cinnamoyl-vulgaroside | 531.31 | pos | 5.89 | 0.5016 | 0.03451 | 0.7827 | 0.2271 | 1.5951 | 0.2332 |
| (S)-N-(4,5-Dihydro-1-methyl-4-oxo-1H-imidazol-2-yl)alanine | 184.07 | neg | 0.81 | 2.4541 | 0.01529 | 1.6381 | 0.2826 | 0.6404 | 0.1182 |
| Gluten exorphin C | 712.40 | neg | 8.29 | 0.5471 | 0.01036 | 0.6482 | 0.07538 | 1.2754 | 0.203 |
| (±)-Pandamarine | 360.20 | neg | 4.92 | 0.501 | 0.03272 | 0.583 | 0.06302 | 1.0686 | 0.8593 |
| 6-Propyltridecane | 290.28 | pos | 6.67 | 1.3604 | 0.00003704 | 1.1762 | 0.08113 | 0.8457 | 0.01914 |
| (R)-2-Hydroxysterculic acid | 331.23 | neg | 7.93 | 1.2589 | 0.000001671 | 0.9494 | 0.2074 | 0.7525 | 1.03E-07 |
| 11Z-hexadecenoic acid | 255.23 | pos | 9.22 | 1.1883 | 0.000009682 | 1.028 | 0.3062 | 0.8526 | 9.6E-07 |
| N-acetyltryptophan | 310.12 | pos | 1.08 | 0.5992 | 0.00004592 | 0.8428 | 0.1041 | 1.4884 | 0.001855 |
| N-Ethyl trans-2-cis-6-nonadienamide | 226.14 | neg | 6.49 | 1.4214 | 0.0006078 | 1.211 | 0.1559 | 0.8248 | 0.0399 |
| 1,6,9-Farnesatriene-3,11-diol | 239.20 | pos | 8.36 | 1.455 | 0.03436 | 0.8461 | 0.5504 | 0.622 | 0.001716 |
| Phenylalanylphenylalanine | 357.12 | pos | 4.30 | 1.6667 | 0.02064 | 0.9162 | 0.7641 | 0.5317 | 0.004625 |
| Minocycline | 456.18 | neg | 9.98 | 0.7231 | 2.357E-07 | 0.9232 | 0.08746 | 1.2926 | 7.16E-07 |
| PI(20:4(5Z,8Z,11Z,14Z)/0:0) | 621.30 | pos | 7.36 | 1.2319 | 0.001649 | 0.9526 | 0.3939 | 0.7961 | 0.000354 |
| LysoPE(0:0/24:0) | 610.41 | neg | 9.91 | 1.2059 | 2.151E-07 | 1.0612 | 0.1177 | 0.8725 | 0.000135 |
| 3-hydroxyhexanoyl carnitine | 270.17 | neg | 6.54 | 1.6091 | 0.01724 | 1.326 | 0.247 | 0.7759 | 0.05195 |
| 3-Hydroxysebacic acid | 183.10 | pos | 4.12 | 0.5066 | 0.009647 | 0.9129 | 0.6887 | 1.6787 | 0.09767 |
| Dictyoquinazol B | 373.14 | neg | 4.79 | 0.6215 | 0.009055 | 0.7776 | 0.1056 | 1.3418 | 0.1176 |
| (E)-2,6-Dimethyl-2,5-heptadienoic acid | 155.11 | pos | 6.25 | 1.3612 | 0.01691 | 1.0575 | 0.721 | 0.7666 | 0.001021 |
| LysoPC(20:1(11Z)) | 594.38 | neg | 9.21 | 1.1285 | 0.000003784 | 1.0173 | 0.5101 | 0.903 | 6.78E-05 |
| Sinapoylputrescine | 275.14 | neg | 5.95 | 0.6268 | 0.01903 | 0.8634 | 0.1192 | 1.1966 | 0.3497 |
| 1alpha-Hydroxyarbusculin A | 301.12 | neg | 6.67 | 1.462 | 0.005542 | 0.9503 | 0.754 | 0.6822 | 0.000127 |
| Hydroxyprolyl-Glutamate | 259.09 | neg | 4.53 | 0.4515 | 0.004077 | 0.7913 | 0.3818 | 1.2436 | 0.5526 |
| PC(18:2(9Z,12Z)/P-16:0) | 786.57 | neg | 11.59 | 1.2462 | 0.0001704 | 1.0321 | 0.6168 | 0.8165 | 0.000543 |
| Lucernol | 329.03 | neg | 4.24 | 1.3756 | 0.00009868 | 1.1416 | 0.3395 | 0.7814 | 0.04914 |
| Lepidimoic acid | 323.10 | pos | 4.63 | 1.5332 | 0.007631 | 1.1164 | 0.5628 | 0.6673 | 0.002403 |
| Ecgonine | 230.10 | neg | 4.10 | 0.7228 | 0.01419 | 0.9899 | 0.9503 | 1.3437 | 0.05847 |
| N-(1-Deoxy-1-fructosyl)valine | 314.10 | neg | 0.82 | 0.3844 | 0.000001501 | 0.9901 | 0.909 | 2.7328 | 8.69E-08 |
| Foeniculoside VIII | 383.14 | neg | 3.82 | 0.5816 | 0.006606 | 0.9689 | 0.8661 | 1.8102 | 0.01617 |
| (24R,25S)-25,26-epoxy-1alpha,24-dihydroxy-27-norvitamin D3 | 417.30 | pos | 8.81 | 2.1465 | 0.0005178 | 1.004 | 0.8279 | 0.4602 | 2.53E-05 |
| Liquiritin | 383.12 | pos | 0.68 | 0.7902 | 2.574E-10 | 0.9953 | 0.635 | 1.2593 | 1.74E-11 |
| 2-O-alpha-D-Galactopyranosyl-1-deoxynojirimycin | 367.15 | pos | 0.86 | 0.8457 | 0.000001322 | 0.9836 | 0.515 | 1.1662 | 1.13E-06 |
| Solerol | 145.05 | neg | 1.71 | 0.5937 | 0.0002779 | 0.9606 | 0.7504 | 1.5939 | 0.00901 |
| Germacrenone | 237.19 | neg | 6.67 | 3.6462 | 0.00003102 | 1.0604 | 0.8552 | 0.2616 | 3.7E-07 |
| N-(1-Deoxy-1-fructosyl)methionine | 312.11 | pos | 1.34 | 0.834 | 0.00001668 | 1.006 | 0.8855 | 1.2042 | 4.54E-05 |
| (1'R)-Nepetalic acid | 183.10 | neg | 5.76 | 1.9143 | 0.001628 | 1.0462 | 0.8933 | 0.5729 | 0.003864 |
| N-(1-Deoxy-1-fructosyl)tyrosine | 378.09 | neg | 3.85 | 0.7497 | 0.0002476 | 0.9935 | 0.8896 | 1.3369 | 6.39E-05 |
| Aspartyl-Arginine | 328.10 | pos | 5.04 | 1.3631 | 0.001847 | 0.9408 | 0.7045 | 0.6628 | 0.000277 |
| 2-(5-Methyl-2-furanyl)piperidine | 210.11 | neg | 4.60 | 0.0001 | 0.002678 | 1.1013 | 0.8403 | 7871.517 | 0.01841 |
| 3-Indolepropionic acid | 190.09 | pos | 5.12 | 1.4426 | 0.00003694 | 0.9897 | 0.8484 | 0.6841 | 6.3E-07 |
| 3''-O-Caffeoylcosmosiin | 577.13 | pos | 5.81 | 4.9195 | 1.459E-12 | 0.904 | 0.5131 | 0.2098 | 2.41E-15 |
| Dihydronaringenin-O-sulphate | 353.03 | neg | 5.03 | 2.8973 | 0.0002412 | 1.0493 | 0.8914 | 0.3145 | 6.21E-06 |

Note: FC: fold change; RT: retention time; M/Z: mass-to-charge ratio; MC: model control group; NC: normal control group; PCFM: prolamin from cooked foxtail millet group. Student’s t test was applied to calculate the significance of metabolite intensities differences among different groups (n=6-7 mice/group).

**Table S2.** Summary of the KEGG pathways influenced by diabetes in mice

| Pathway_ID | Pathway Desciption | Impact-value | *p* value_ |
| --- | --- | --- | --- |
| map00500 | Starch and sucrose metabolism | 0.421313 | 0.019365 |
| map00830 | Retinol metabolism | 0.262887 | 0.101852 |
| map00380 | Tryptophan metabolism | 0.084091 | 0.254501 |
| map00592 | alpha-Linolenic acid metabolism | 0.07173 | 0.013699 |
| map00564 | Glycerophospholipid metabolism | 0.069878 | 0.002844 |
| map00730 | Thiamine metabolism | 0.056657 | 0.160773 |
| map00140 | Steroid hormone biosynthesis | 0.048342 | 0.091887 |
| map00052 | Galactose metabolism | 0.03349 | 0.002524 |
| map00260 | Glycine, serine and threonine metabolism | 0.016527 | 0.231836 |
| map00120 | Primary bile acid biosynthesis | 0.008193 | 0.228383 |
| map00270 | Cysteine and methionine metabolism | 0.003576 | 0.257531 |
| map00010 | Glycolysis / Gluconeogenesis | 0.002206 | 0.000812 |
| map00030 | Pentose phosphate pathway | 0 | 0.019365 |
| map00520 | Amino sugar and nucleotide sugar metabolism | 0 | 0.023438 |
| map00591 | Linoleic acid metabolism | 0 | 0.079924 |
| map00600 | Sphingolipid metabolism | 0 | 0.122607 |
| map00590 | Arachidonic acid metabolism | 0 | 0.194758 |
| map00310 | Lysine degradation | 0 | 0.241867 |
| map00360 | Phenylalanine metabolism | 0 | 0.245104 |
| map00051 | Fructose and mannose metabolism | 0 | 0.248288 |
| map00970 | Aminoacyl-tRNA biosynthesis | 0 | 0.248288 |
| map00040 | Pentose and glucuronate interconversions | 0 | 0.25142 |
| map00350 | Tyrosine metabolism | 0 | 0.271933 |
| map00524 | Neomycin, kanamycin and gentamicin biosynthesis | 0 | 0.310251 |
